# BuRNN: Buffer Region Neural Network Approach for Polarizable-Embedding Neural Network/Molecular Mechanics Simulations

**DOI:** 10.1021/acs.jpclett.2c00654

**Published:** 2022-04-25

**Authors:** Bettina Lier, Peter Poliak, Philipp Marquetand, Julia Westermayr, Chris Oostenbrink

**Affiliations:** † Institute for Molecular Modeling and Simulation, Department of Material Sciences and Process Engineering, University of Natural Resources and Life Sciences, Vienna, Muthgasse 18, 1190 Vienna, Austria; ‡ Department of Chemical Physics, Institute of Physical Chemistry and Chemical Physics, Faculty of Chemical and Food Technology, Slovak University of Technology in Bratislava, Radlinského 9, 812 37 Bratislava, Slovakia; § Institute of Theoretical Chemistry, University of Vienna, Währingerstraße 17, 1090 Vienna, Austria; ∥ Department of Chemistry, University of Warwick, Gibbet Hill Road, Coventry CV4 7AL, U.K.

## Abstract

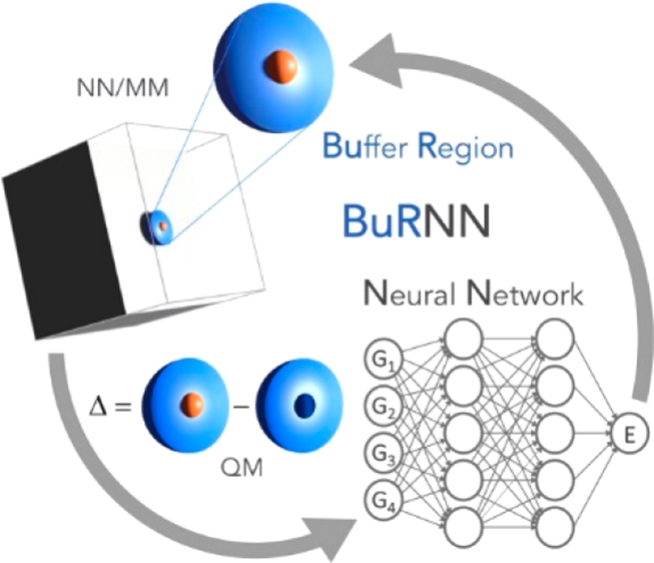

Hybrid quantum mechanics/molecular mechanics (QM/MM) simulations have advanced the field of computational chemistry tremendously. However, they require the partitioning of a system into two different regions that are treated at different levels of theory, which can cause artifacts at the interface. Furthermore, they are still limited by high computational costs of quantum chemical calculations. In this work, we develop the buffer region neural network (BuRNN), an alternative approach to existing QM/MM schemes, which introduces a buffer region that experiences full electronic polarization by the inner QM region to minimize artifacts. The interactions between the QM and the buffer region are described by deep neural networks (NNs), which leads to the high computational efficiency of this hybrid NN/MM scheme while retaining quantum chemical accuracy. We demonstrate the BuRNN approach by performing NN/MM simulations of the hexa-aqua iron complex.

Molecular dynamics (MD) simulations are powerful tools for studying the dynamics of systems consisting of hundreds of thousands of atoms. The energy of the system can be described fully classically by a molecular mechanics (MM) force field, by a quantum mechanical (QM) method, or by a hybrid quantum mechanics/molecular mechanics (QM/MM) technique. The latter approach is very powerful, as it enables an accurate description of a small important part of a system at the appropriate level of quantum chemistry, while the remainder is treated by MM to simulate large system sizes at relevant time scales.^1^

In QM/MM approaches, the electrostatic coupling between the partitioned regions can be treated with different levels of mutual interaction, i.e., embedding schemes.^2−5^ Mechanical embedding is the simplest and least accurate approach. Interactions are described via classical point charges only. In contrast, electrostatic embedding is physically better motivated, as the QM system experiences the MM charge distribution being embedded in the QM Hamiltonian. However, QM particles see MM particles as fixed point charges, which neglects polarization in the MM region. To account for polarization effects in the MM region, as well, polarizable force fields can be used.^6,7^

Independently of the scheme, all QM/MM methods are limited by high computational costs of the quantum calculation and issues at the interface, such as overpolarization.^8^ Particularly prone to such artifacts are boundaries that cross covalent bonds, although a careful choice of the bond splitting scheme can alleviate them.^4^ Furthermore, discrepancies between the forces derived for the QM and MM region can lead to artifical crowding or depletion at the interface, when particles are allowed to change character during a simulation. Several approaches have been proposed to address boundary artifacts either by introducing an intermediate region^9,10^ or by restricting the boundary transition.^11^

Alternatively, the whole system can be treated using machine-learned interatomic potentials based on ab initio data.^12^ Machine learning (ML) is especially effective for MD simulations as it can learn the relation between a descriptor, i.e., the structure of a system, and a targeted output, i.e., energies and forces, with the accuracy of the reference method, but much lower computational costs. Such ML potentials are available for specific materials at different levels of theory.^13−17^ However, universal ML potentials for more complex systems, such as biomolecules, still pose a challenge and are limited by the computational expenses of the reference calculations.^2,12^

Very recently, ML potentials have been combined with QM/MM concepts and were shown to be powerful for, e.g., calculating free energies or transition paths.^18−24^ However, these approaches are complicated as ML models need to capture the effects of the environment (MM region) even though only the QM region has to be learned. The introduction of a cutoff, up to which the MM region is included, has emerged as a solution.^21,22,25^ One example is FieldSchNet,^20^ which circumvents this problem by sampling the environment while keeping the QM region fixed. This model has been shown to be powerful in predicting spectra and chemical reactions with neural networks (NNs) using electrostatic embedding but requires extended sampling. Due to the nature of electrostatics, artifacts at the interfaces are not reduced in the aforementioned approaches.

To circumvent boundary problems and with the aim of avoiding extensive force field parametrizations, we propose an alternative approach. We introduce an additional buffer region that experiences full electronic polarization by the inner QM region. The buffer region is described at the QM and MM levels. Effectively, the interactions with the QM region are calculated entirely at the QM level while the interactions with the MM region are described at the MM level. Within the buffer region, the interactions are a combination of MM interactions and the effect of the QM region on the electronic degrees of freedom of the buffer region. While this approach minimizes the artifacts that arise from mixing two levels of theory, it comes at considerable computational costs as two QM calculations are required. By using ML to describe QM-derived energy surfaces, an elegant solution emerges. In this work, we train deep NNs to directly predict the difference between the two required QM calculations. Thus, this scheme automatically includes the mutual influence of the QM and the buffer region, without the need for additional external forces in the ML setup.^20^ The calculation of all relevant energies in a simulation can efficiently be done with a single evaluation of the NN. Due to the fact that interaction energies are often easier to learn with NNs than potential energies, outstanding accuracy can be achieved with mean absolute errors in the range of a few kilojoules per mole. This range is well below the often-desired chemical accuracy of machine learning models and is independent of the size of the inner region. We refer to this NN/MM approach as a buffer region neural network (BuRNN). Although schemes like ONIOM with different regions exist, this approach is, to the best of our knowledge, novel.

The BuRNN approach partitions a system into an inner region , a buffer region , and an outer region  (Figure 1). The total potential energy, *V*_tot_, contains the QM energy of the inner and buffer region, , and the MM energy coupling of the outer region to all other regions, . The buffer region is calculated at both levels of theory. The difference of the two buffer terms, , is included in *V*_tot_ and helps to smooth the transition between the QM energy of the inner region and the MM energy of the outer region. In addition, artifacts in the electronic degrees of freedom at the outer edge of the buffer region will largely cancel in the difference . Further details are discussed below. Adding all terms for the total potential energy together leads to
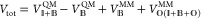
1

**Figure 1:**
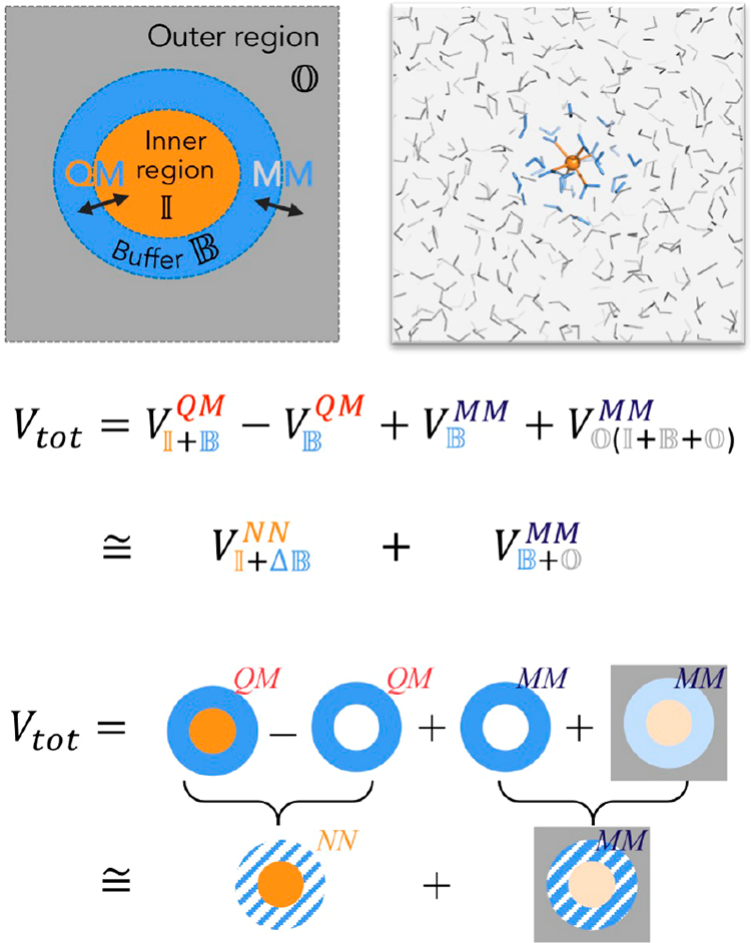
Scheme of the BuRNN approach, which distinguishes three regions: (1) inner region  (orange), which is described entirely by quantum mechanics (QM), (2) buffer region  (blue), which is described by both QM and molecular mechanics (MM), and (3) outer region  (gray), which is described entirely by a classical MM force field.

The subscripts for the potential energy denote the calculated region, and the superscripts the method. Even though interactions in QM are not pairwise additive, it is instructive to consider them as hypothetical pairwise interactions within or between regions. They are indicated with a comma-separated subscript.  can then be separated into three terms, i.e., the energy that results from interactions within the inner region, , between the inner and buffer region, , and within the buffer region, :
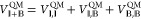
2

The potential energy of the buffer region, , is separately calculated at the QM level, as well, and is denoted as . This should emphasize that the inner region is not part of this particular calculation and term.  as a hypothetical pairwise interaction within the buffer region as used in eq 2, though, includes the influence of the inner region on the buffer. The difference of the two terms can be seen as the polarization of the buffer, which is denoted as :
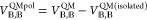
3

The energy of the buffer is also described at the MM level, . If this term agrees with QM exactly, it will cancel with . The outer region with all involved interactions , , and  is treated at the MM level, but with partial charges of the inner and buffer regions derived from the QM calculation of the inner and buffer region together. Hence, it also includes a representation of the polarization of the inner and buffer regions:
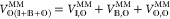
4

The total energy in terms of hypothetical interaction energies can then be written as
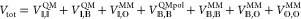
5

Thus, BuRNN ensures that the interactions within and between neighboring regions are computed at the appropriate levels. One of the benefits is that any artifacts in the electronic degrees of freedom will cancel in the difference , as mentioned above. This is true with the assumption that these artifacts are due to the interface with the outer region and that the relevant polarization of the buffer region predominantly takes place at the interface between the inner and buffer regions. More specifically, artifacts in the QM calculation may arise in the electron density at the outer boundary of the buffer region, which differs from a solvated boundary. However, these artifacts will cancel in the difference as it will be very similar in both QM terms. Any remaining artifacts potentially arising at the interface between the buffer and the outer region are, furthermore, relatively far from the inner region of interest.

The interactions between  and between  are computed using a mechanical-embedding scheme with charges assigned from the QM calculation, which is appropriate because of the large distances between the inner and outer regions. The direct electronic influence on the inner region due to the outer region will be relatively small, and the interaction is largely electrostatic. The remaining artifacts may arise from the fact that particles moving from the outer region into the buffer region switch from a mechanically embedded MM interaction to a full QM interaction with the inner region. Also in this case, the interaction is a relatively long distance from the inner region, where the  interaction will be largely electrostatic in nature, such that these artifacts can be expected to be small.

Despite the accuracy and benefit of this scheme, the burden lies in the high computational costs that remain because two computationally expensive QM calculations are required. To overcome this limitation, we describe the first two terms of eq 1 directly using a deep NN:
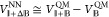
6which is equal to
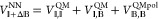
7

It now becomes clear that the introduction of a buffer region into the BuRNN is akin to a polarizable embedding with the polarization described at the full QM level. Thus, the NN represents the full interactions within the inner region, the interactions between the inner and buffer regions, and the polarization of the buffer region due to the inner region in a single term, . The Δ sign is used to emphasize that the BuRNN essentially includes a Δ learning,^26^ bringing interactions of the buffer region from the MM to the QM level. The total BuRNN energy can finally be rewritten as
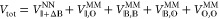
8

All MM terms can be computed classically from a single call to the force field. The workflow of an NN/MM BuRNN simulation is illustrated in Figure 2. The training data set is based on QM calculations and can be generated via sampling from MD simulation snapshots of the targeted system and extended using adaptive sampling.^27^ The training set generation and sampling of initial data are explained in detail in sections S1.1 and S1.2 of the Supporting Information. We employ NNs to predict interaction energies, interaction forces, and charges to carry out NN/MM simulations. As NN models, we use SchNet,^28,29^ a deep convolutional continuous-filter NN, that was adapted to allow for charge fitting. A full description of the NN models, including learning curves, model accuracy, and hyperparameter optimization, can be found in section S1.3. The mean absolute error assessed from five independently trained NN models is 1.7 ± 0.3 kJ/mol for energies, 8.4 ± 0.4 kJ mol^–1^ nm^–1^ for forces, and 0.027 ± 0.001 au for partial charges. Models trained on a larger inner region are comparable in accuracy, as the NN models are always trained on the whole system, i.e., the inner and buffer regions, which have >50 atoms. For all of the outputs, these are very small errors, well below the chemical accuracy defined as 1 kcal/mol in recent (machine learning) studies.^26,30^ We have implemented the BuRNN approach in the GROMOS simulation software^31^ (see section S1.3). Importantly, we generate the training set and conduct MD simulations by applying two initially trained NNs, A and B. In adaptive sampling, their prediction differences can be used to assess the reliability of NN models during the MD simulation. Whenever the NN predictions for interaction energies are similar, i.e., when , predictions are deemed accurate and the simulation is continued. If predictions start to diverge from each other and exceed a predefined threshold, additional reference QM calculations (and ) are performed for relevant configurations and added to the training set. In this work, we carried out four rounds of adaptive sampling, i.e., until dynamics could be run up to 1 ns without further interruptions. By replacing QM calculations with NNs during MD simulations, the BuRNN reduces the computational costs considerably and enables long time scales while retaining high accuracy.

**Figure 2:**
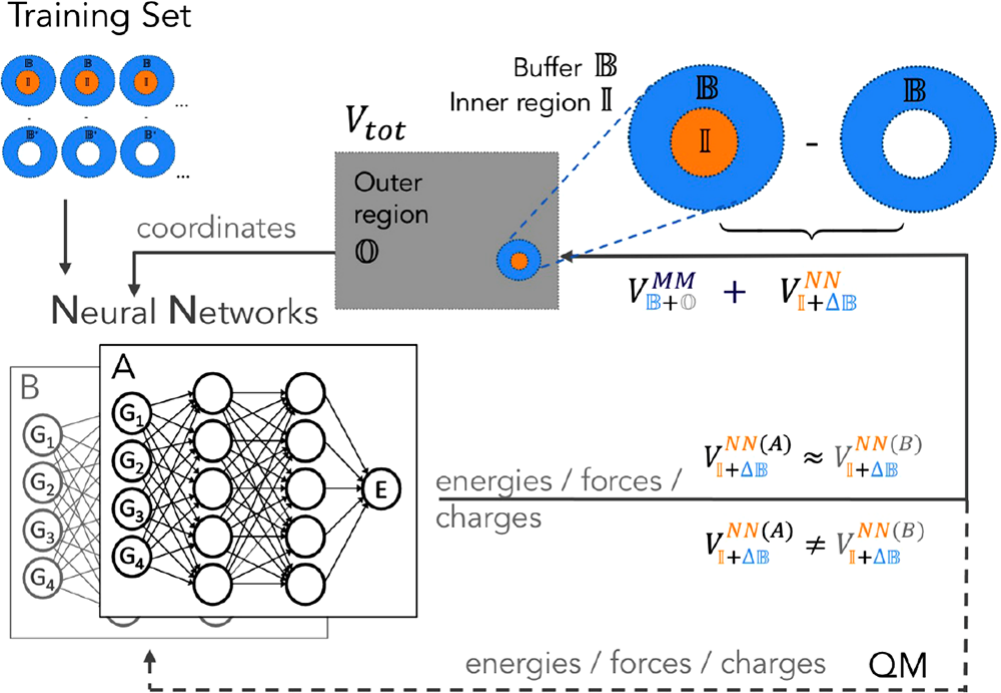
Process of a BuRNN simulation that includes adaptive sampling. At every *x*th time step during MM, two neural networks (A and B) are compared. When predictions diverge, the training set is expanded by additional QM calculations.

Here, we demonstrate the use of the BuRNN for the hexa-aqua iron [Fe(H_2_O)_6_]^3+^ complex in water as a model system. This system has the advantage of being relatively simple for testing our approach, but the classical description of transition metal interactions is notoriously difficult, which makes it a good use case of the BuRNN for simulating this system. Especially challenging is the coordinative bond between Fe and O as it is somewhat between a covalent bond and an ionic bond and is often addressed with specialized force fields.^32,33^

In our test case, the Fe^3+^ ion comprises the inner region, and water molecules up to 0.5 nm are treated as the buffer region, which roughly accounts for the first two solvation shells, where the first solvation shell is expected to be formed by the hexacoordinated waters. During extensive MD simulations (10 ns), the water molecules are freely diffusing between the buffer and outer regions, smoothly switching interactions between the NN (QM) level and the MM level of theory. BuRNN simulations are validated using experimental data and are compared to QM/MM simulations of the Fe(H_2_O)_6_^3+^ complex (QM region) in classical SPC water and two distinct fully classical descriptions. In addition, we performed BuRNN simulations using a larger inner region that additionally comprises the first solvation shell.

To validate the method, we first look at the Fe–O radial distribution functions (RDF) *g*(*r*) in Figure 3a. All simulations show a distinct peak at ∼0.20 nm, corresponding to the first coordinative solvation shell of mostly six water molecules, which is slightly narrower and more pronounced in the classical description (blue curve). In contrast, the second solvation shell is more pronounced in the BuRNN (orange curve) and corresponds to an average of 12.7 water molecules. It shows a maximum at ∼0.41 nm, while the MM simulation shows a broader peak with a maximum at 0.40 nm. Simulations with a larger inner region yield almost identical results (Figure S4). In the QM/MM simulation with electrostatic embedding, the second shell also has a maximum at 0.40 nm (gray curve), while a QM/MM simulation with mechanical embedding leads to a maximum at 0.41 nm (Figure S4), as in the BuRNN. Experimentally, it was found at 0.415 nm and comprises 12 water molecules, hence agreeing well with our simulations.^34^ We have also performed simulations using the 12–6–4 Lennard-Jones potential^35^ and the SPC/E water model^36^ and found that the *g*(*r*) shows an additional peak at 0.31 nm, representing one additional molecule pushing into the first solvation shell (Figure S4). The transition at 0.5 nm in the RDF obtained with the BuRNN is smooth and does not show any artifacts. This is remarkable as the buffer region ends and the water molecules beyond this distance interact completely according to a pure MM description.

**Figure 3:**
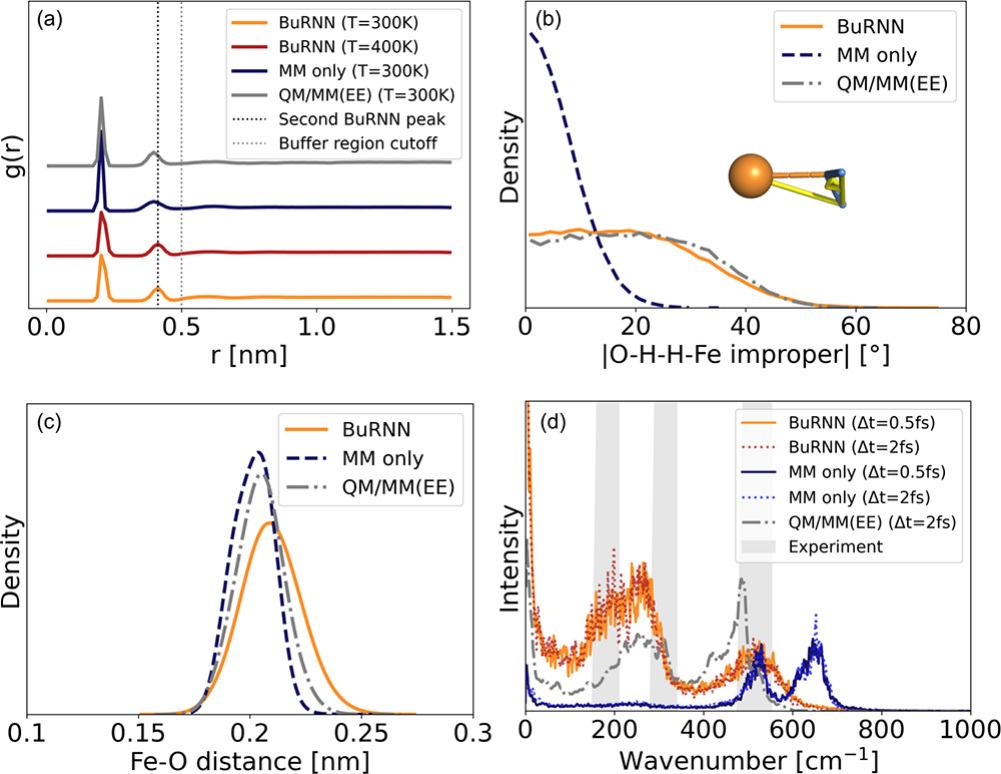
Coordination of Fe^3+^ by water molecules with BuRNN simulations and when using MM only. (a) Radial distribution function for BuRNN at temperatures of 300 and 400 K, with MM only and a QM/MM simulation using electrostatic embedding (EE). The dashed lines indicate the second BuRNN peak and the cutoff used to define the buffer region. (b) Probability distribution of the O–H–H–Fe improper dihedral. (c) Distribution of the Fe–O distance. (d) Power spectrum of the Fe–O coordinative bond for different simulations. Experimental data were taken from refs (50−52).

To investigate the robustness of the BuRNN, we performed MD at different temperatures (Figure S5) and show the RDF obtained at 400 K in Figure 3a. As one can see, there is a slight smoothing between 0.5 and 1.0 nm due to the increased level of thermal motion, but the BuRNN simulation remains stable. The two NN models deviate on average by 0.39 ± 0.02 kJ/mol.

In addition, we sought to investigate the propensity of the BuRNN to describe water exchange. Hence, we used umbrella sampling^37^ to pull a water molecule away from the complex and observed the spontaneous exchange of this water molecule with another (see Supporting Movie S1). The energy predictions and MD simulations are stable during this process. In the regular simulations, the hexacoordination is stably maintained. Water molecules in the second solvation shell (within the buffer region) readily exchange with water molecules from the outer region. All water molecules (786 molecules) visit the buffer region at least once during the simulation, with an average lifetime of 14.4 ps. This agrees with estimates from NMR experiments that determine a lifetime that is below their resolution limit of 100 ps.^38^ We further computed the self-diffusion rate for BuRNN and MM only simulations and found values of 0.98 × 10^–5^ and 0.92 × 10^–5^ cm^2^/ps, respectively. Both approaches overestimate the diffusion constant compared to experimental estimates of 0.55–0.68 × 10^–5^ cm^2^/ps,^38−41^ in line with the observation that bulk SPC (simple point charge) water has a diffusion constant that is slightly too large.^42^

Figure 3b shows the distribution of the O–H–H–Fe improper dihedral angles defining the co-planarity of the iron and a water molecule. A value of 0°, which is predominant in pure MM simulations, implies that the water molecule and the Fe^3+^ ion are in the same plane. Larger values as observed for the BuRNN with a mean angle of 19.3° and for QM/MM simulations (mean angle of 20.3°) indicate a more tetrahedral arrangement in which the iron interacts with the lone pairs on the oxygen. For comparison, a BP86-D3/def2-TZVP/COSMO estimate for this angle in [Fe(H_2_O)_18_]^3+^ lies at 16°.^43^

MD simulations are further compared by the geometries visited during the simulations. Figures 3c shows radial distances between the Fe and O that agree well with the range of experimental estimates for the Fe–O bond lengths of 0.199–0.210 nm.^34,44−50^ O–Fe–O angles are almost identical among the BuRNN, QM/MM, and “MM only” and reflect angles expected for an octahedral arrangement (peaking at around 90° and 175°). Figure 3d shows that there are clear differences for the frequencies at which the Fe–O bonds vibrate, implying that the Fe–O interaction is indeed not captured well by a purely classical description. In the QM/MM simulations and when using the BuRNN, the vibrations take place at lower frequencies and are in better agreement with experimental bands observed at ∼180, ∼310, and ∼500 cm^–1^.^50−52^ The frequencies obtained with quantum chemistry are better aligned with experiment and the BuRNN than with pure MM (Figure S4b), while those obtained from 12–6–4 Lennard-Jones potential simulations are even higher than those observed with the simple MM only approach (Figure S4).

In this work, we have introduced the BuRNN, a buffered region neural network NN/MM scheme, as an alternative to QM/MM simulations that experiences full electronic polarization by the inner QM region. The BuRNN minimizes artifacts at the interface between regions by ensuring that interactions that go over boundaries are treated at a consistent level of theory. Inconsistencies at the edges of the buffer (i) can be expected to cancel in the difference between two QM terms and (ii) are far removed from the inner region. These advantages come at the cost of an additional QM calculation, which is elegantly solved by training NNs directly on the energy difference. A single evaluation of the NN is required to evaluate the energies, and a second NN is used to derive charges for full mutual polarization. The BuRNN allows fast hybrid NN/MM simulations and has the advantage of being applicable to any system and usable with any molecular ML model.

We have demonstrated the use of the BuRNN by realistic simulations of hexa-aqua iron in water. This shows that it can be applied for metal–ligand interactions without the need for additional force field parameters. The good agreement and high stability of BuRNN for long time-scale MD simulations, including external perturbation, such as changing temperature or forces that lead to water exchange, make our method very promising for future application in the simulation of more complex systems.

## Supporting Information Available

Computational details for the quantum calculations, training set generation, training and validation of the neural networks and simulation settings, and further analyses of simulations with alternative settings (QM/MM with mechanical embedding, classical with a 12–6–4 Lennard-Jones potential, BuRNN with alternative charge distributions, BuRNN at higher temperatures, and BuRNN with a larger inner region) (PDF)
Supporting Movie S1 (MP4)
Transparent Peer Review report available (PDF)
