# Supplementary material for: BuRNN: Buffer Region Neural Network Approach for Polarizable-Embedding Neural Network/Molecular Mechanics Simulations
Source: J Phys Chem Lett. 2022 Apr 25;13(17):3812–8. doi: 10.1021/acs.jpclett.2c00654 (PMC9082612; doi:10.1021/acs.jpclett.2c00654)
Supplement: Supplementary file 1 — jz2c00654_si_001.pdf [file jz2c00654_si_001.pdf]

Supplementary Information for:

BuRNN: Buffer Region Neural Network Approach for Polarizable-Embedding Neural Network / Molecular Mechanics Simulations

*Bettina Lier,[#a] Peter Poliak,[#a,b] Philipp Marquetand,[c] Julia Westermayr[*d] and Chris Oostenbrink[*a]*

a. *Institute for Molecular Modeling and Simulation, Department of Material Sciences and Process Engineering, University of Natural Resources and Life Sciences, Vienna. Muthgasse 18, 1190 Vienna Austria*

b. *Department of Chemical Physics, Institute of Physical Chemistry and Chemical Physics, Faculty of Chemical and Food Technology, Slovak University of Technology in Bratislava, Radlinského 9, 812 37 Bratislava, Slovakia*

c. *Institute of Theoretical Chemistry, University of Vienna, Währingerstraße 17, 1090 Vienna, Austria*

d. *Department of Chemistry, University of Warwick, Gibbet Hill Road, CV4 7AL Coventry, UK.*

# Authors contributed equally

** Corresponding authors: julia.westermayr@warwick.ac.uk and chris.oostenbrink@boku.ac.at*

Contents

S1 Computational Details 2
S1.1 Quantum Chemical Calculations 2
S1.2 Data set generation 3
S1.3 Neural Networks 5
S1.3.1. Neural network training 5
S1.3.2 Hyperparameter optimization 7
S1.3.2. Neural network accuracy 7
S1.4 Molecular Dynamics Simulations 10
S2 BuRNN simulations 12
S2.1 BuRNN forces 12
S2.2 Observed structure 14
S2.3 BuRNN is robust with respect to higher temperatures 16

S1 Computational Details

S1.1 Quantum Chemical Calculations

Total electronic energies and gradients of the training data points were all calculated in the Gaussian 16 program package[1] using spin-unrestricted density functional theory (DFT) in the gas phase. The iron-containing structures were calculated in the sextet state with a net charge of +3. Based on the benchmark calculations of iron complexes,[2] the OPTX exchange[3] with PBE correlation functional[4] (OPBE) were used in def2SVP basis set.[5] The self-consistent field (SCF) procedure was performed using the 'yqc' option. The energy minimisations were carried out by the default Berny optimisation algorithm. The spin contamination of the sextet species was below 10^{-5} a.u.. The use of the input orientation was enforced using the 'nosymm' keyword. Partial atomic charges were obtained from single-point calculations on the inner+buffer region by fitting to the electrostatic potential using the Merz-Singh-Kollman scheme.[1, 6-8] The partial atomic charges in the water droplet of 1.0 nm diameter calculated by this approach are very similar to the charges of the SPC water model, therefore further adjustment was not needed. Note that the fitting procedure bears the risk that the partial charges of the inner core are

insensitive to the electrostatic potential further out. However, due to the large central charge on the iron ion, this problem does not occur. See Figure S1 for the distributions of the partial charges on Fe, O and H, in the training set, which are very stable. Average charges in the data set were 1.29, -0.73 and 0.41 for the iron, oxygen and hydrogen atom, respectively. Panel b shows the atomic charges as a function of the distance to Fe^{3+}. The outliers in the oxygen charges come from configurations in the training set in which the water molecules are oriented with their hydrogen atoms towards the Fe^{3+}. We note that use of gas-phase calculation for energies, gradients and partial charges is crucial as the buffer region in BuRNN interacts explicitly with the water in the outer region and the effect of, e.g., an implicit solvent would lead to double counting of solvation effects.

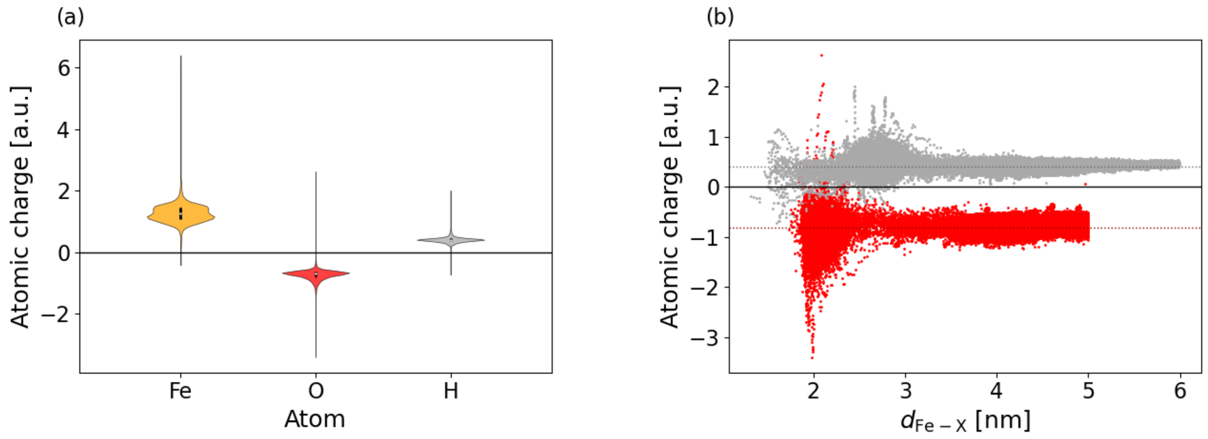

Figure S 1: (a) Partial atomic charges of the data set and (b) their dependence on the distance to the Fe atom for oxygen (red) and hydrogen (grey). Dotted lines represent corresponding atomic charges in the SPC water model.

S1.2 Data set generation

The initial data set was generated from a molecular dynamics (MD) simulation (pure molecular mechanics description) set up as described in the Methods section S1.4. From an 8-ns MD simulation, snapshots were selected every 4 ps. Water molecules with the oxygen atom further than 0.5 nm from the iron atom were discarded, yielding Sa total of 2,000 configurations, for which single point QM calculations of the inner+buffer and for the buffer region alone were performed in gas-phase, as further described in section S1.1. After training on the initial training set, short MD simulations and optimizations with the BuRNN approach were performed on a system containing inner, buffer, and outer regions. These simulations did not lead to stable hexa-coordinated water configurations, but another 100 configurations were generated. To enhance accuracy close to the minimum, the 21 lowest

energy configurations from the BuRNN simulation were extracted and minimized at the QM level. From these minimization trajectories, about 4,000 new configurations were extracted. The initial model was retrained on the updated data set, which led to much more stable BuRNN simulations, but we still observed irregular behaviour in the coordination sphere and the boundary region and large model deviations during simulations. Therefore, we postprocessed all configurations by a) applying SHAKE to ensure that the conformations of the water molecules adhered to the SPC configuration and b) removing any water molecules that in the QM optimization moved outside of the 0.5 nm cutoff. This added the latest 4,000 configurations a second time to the training set.

The NNs that were trained after this step led to stable simulations in the BuRNN approach, with differences between two independent NNs on the order of 10 kJ/mol. We next generated one additional set of conformations a) by picking the conformations for which the disagreement between the two independent NNs was largest during a BuRNN simulation (1,000 configurations), b) by running a BuRNN MD simulation with applying a biasing potential derived from the disagreement between the two independent NNs to sample the undertrained conformational space as

$$V_{\text{bias}} = \begin{cases} \frac{1}{4} K_{bias} \left[\left(V_{\mathbb{I}+\Delta\mathbb{B}}^{NN(A)} - V_{\mathbb{I}+\Delta\mathbb{B}}^{NN(B)} \right)^2 - {V_{val,thresh}}^2 \right]^2, & V_{\mathbb{I}+\Delta\mathbb{B}}^{NN(A)} - V_{\mathbb{I}+\Delta\mathbb{B}}^{NN(B)} > V_{thresh} \\ 0, & V_{\mathbb{I}+\Delta\mathbb{B}}^{NN(A)} - V_{\mathbb{I}+\Delta\mathbb{B}}^{NN(B)} \leq V_{thresh} \end{cases} \quad \text{S1}$$

where K_{bias} was set to –0.01 kJ^{-3} mol^{3}, V_{thresh} to 1.0 kJ/mol^{-1} and $V_{\mathbb{I}+\Delta\mathbb{B}}^{NN(B)}$ is a predicted energy from the second NN model (500 configurations), c) selecting conformations from a simulation in which one water molecule was artificially pulled away from the Fe^{3+} (300 configurations). This was done to ensure that the NNs learned energies and partial charges with a water molecule between the first and second solvation shell as well. Configurations were compared by energies, forces and spin contamination and obvious outliers were discarded leading to an overall training set of about 11,000 configurations, of which 9,500 were used for training and the rest for testing.

We emphasize that the procedure outlined above was the result of a continuous process to obtain a sufficiently large training set, such that we could test the BuRNN approach and not the training. For any further applications we are confident that generation of an initial training set that is SHAKEN and

filtered up to the buffer region, followed by an adaptive sampling scheme to generate additional conformations as needed will be sufficient.

S1.3 Neural Networks

S1.3.1. Neural network training

Deep NNs used in this work are based on the continuous-filter convolutional NN SchNet,[9, 10] which is a message-passing NN that learns the molecular descriptor in addition to its relation to target properties. In this work, we use SchNet to train on the interaction energies, $V^{NN}_{\mathbb{I}+\Delta\mathbb{B}}$ (see equations 7 and 8 in the main text) and the corresponding interaction forces, $\boldsymbol{F}^{NN}_{\mathbb{I}+\Delta\mathbb{B}}$, calculated as derivatives of the NN potentials:

$$\boldsymbol{F}^{NN}_{\mathbb{I}+\Delta\mathbb{B}} = -\frac{\partial\, V^{NN}_{\mathbb{I}+\Delta\mathbb{B}}}{\partial \boldsymbol{R}}. \qquad \text{S2}$$

$\boldsymbol{R}$ denotes the atomic positions of all atoms within the QM region (inner and buffer region) and we used a shorthand notation to indicate the derivatives with respect to each of the elements of the vector.

The loss function used for training contains both terms, i.e., interaction energies and interaction forces (see equations S2-S4). The training set contained outliers, i.e., unfavourable structures, e.g., 1-4 coordinated aqua-Fe-complexes, with large energies and forces (but not necessarily large interaction energies and interaction forces). To allow a training of a diverse data set including such "outliers", we used a variant of the smooth L_1 loss function, which switches from L_2 to L_1 whenever a data point is deemed to be an outlier. The loss functions used for training energies and forces are given below in equations S3 and S4.

$$L_2 = t\left\|V^{NN}_{\mathbb{I}+\Delta\mathbb{B}} - \left(V^{QM}_{\mathbb{I}+\mathbb{B}} - V^{QM}_{\mathbb{B}}\right)\right\|^2 + (1-t)\left\|-\frac{\partial V^{NN}_{\mathbb{I}+\Delta\mathbb{B}}}{\partial \boldsymbol{R}} + \left(\frac{\partial V^{QM}_{\mathbb{I}+\mathbb{B}}}{\partial \boldsymbol{R}} - \frac{\partial V^{QM}_{\mathbb{B}}}{\partial \boldsymbol{R}}\right)\right\|^2 \qquad \text{S3}$$

$$L_1 = t\left|V^{NN}_{\mathbb{I}+\Delta\mathbb{B}} - \left(V^{QM}_{\mathbb{I}+\mathbb{B}} - V^{QM}_{\mathbb{B}}\right)\right| + (1-t)\left|-\frac{\partial V^{NN}_{\mathbb{I}+\Delta\mathbb{B}}}{\partial \boldsymbol{R}} + \left(\frac{\partial V^{QM}_{\mathbb{I}+\mathbb{B}}}{\partial \boldsymbol{R}} - \frac{\partial V^{QM}_{\mathbb{B}}}{\partial \boldsymbol{R}}\right)\right| \qquad \text{S4}$$

The tradeoff, t, is used to weigh forces and energies during training and was sampled between 0.001 and 100. The optimal value was found to be 0.05. Note that we train forces and not gradients, hence the negative sign.

Atomic partial charges

To describe atomic partial charges, another SchNet model was trained on the full QM region (inner + buffer region). This calculation could be performed on the already converged orbital coefficients of the inner+buffer region, making the additional computational costs negligible and keeping the QM calculations at two.

To allow training of atomic partial charges, the output modules of SchNet had to be adapted such that all atomic partial charges could be learned by one NN. Each atom described in its chemical and structural environment by the NN gave rise to a corresponding atomic partial charge. Therefore, the last pooling layer, which usually sums or averages over all atomic contributions for a target property, was removed. During the simulations, the predicted charges were adjusted to sum up to +3 exactly by homogeneously distributing any charge deficit or surplus (typically 5%) over all particles in the inner and buffer regions.[11]

In contrast to interaction energies and interaction forces, the atomic partial charges, q_a, with a indicating an atom in the whole system containing N_a atoms, were modelled using an L_2 loss:

$$L_2^{charges} = \sum_a^{N_a} \left\| q_{\mathrm{a},\mathbb{I}+\mathbb{B}}^{NN} - q_{\mathrm{a},\mathbb{I}+\mathbb{B}}^{QM} \right\|^2. \qquad \text{S5}$$

Using a smooth L_1 function instead of the L_2 loss did not improve training. As can be seen, multiple values are treated in one NN.

S1.3.2 Hyperparameter optimization

Hyperparameters were assessed for both, interaction energy and interaction force models and atomic partial charge models separately. A random grid search was applied to obtain optimal model parameters. Besides default parameters, we use a cutoff of 1.0 nm and a batch size of 8. The network parameters were validated on a random grid using a training:validation:test split of approximately 8:1:1 and an intermediate training set of 7,900 data points. As SchNet is a message passing neural network (NN), which automatically generates a tailored representation for a given system, it can be seen as a connection of two NNs. The size of the network that models the descriptor based on the structural inputs and elemental charges is defined by 6 interaction layers, 256 features to represent the atoms and 50 Gaussian functions. The number of Gaussian functions was set to 100 for modelling atomic partial charges. The interaction layers were sampled from 4-8, the features from 128-512, and the number of Gaussian functions placed on each atom from 25-200. The learning rate was varied between 0.001 and 0.00001, whereas the default of 0.0001 was most appropriate for training the final data set using 9,500 data points for training. A larger learning rate up to 0.001 was used when training smaller training set sizes. The hidden layers to map the descriptor to the output energies was kept at 3 with more layers not improving training.

S1.3.2. Neural network accuracy

Training directly on interaction energies and interaction forces has several advantages compared to training on the separate terms arising from the inner+buffer region and the buffer region alone. On one hand, our setup allows for higher accuracy and consequently, better data efficiency, as the interaction energies span a smaller energy window than the total energies of the inner+buffer region and the buffer region. While the mean absolute error (MAE) on total energies and forces for models trained on 9,500 data points is in the range of 500 kJ/mol and 5,000 kJ/mol/nm, respectively (resulting in an error of around 700 kJ/mol and 7,000 kJ/mol/nm for interaction energies and interaction forces, respectively), training directly on interaction energies and interaction forces leads to errors about 100-1000 times smaller, i.e., 1.7 ± 0.3 kJ/mol and 8.4 ± 0.4 kJ/mol/nm, respectively. The MAEs and root mean squared errors (RMSE) on a holdout test set for models trained on interaction energies and interaction forces

are shown in Table S1. MAEs and RMSEs of 5 independently trained models for partial charges on 9,500 data points are shown in Table S2.

Table S1: Mean absolute error (MAE) and root mean squared error (RMSE) for energies and forces of 5 models trained on 9,500 data points corresponding to Fig. S1 (a-d).

Neural Network Model	Energy MAE (RMSE) [kJ/mol]	Forces MAE (RMSE) [kJ/mol/nm]
**Model 1**	1.73 (8.32)	8.35 (21.29)
**Model 2**	2.17 (7.94)	9.05 (26.16)
**Model 3**	1.51 (8.68)	7.94 (23.02)
**Model 4**	1.60 (5.55)	8.68 (24.17)
**Model 5**	1.46 (4.63)	8.08 (19.65)

Table S2: Mean absolute error (MAE) and root mean squared error (RMSE) for charges of 5 models trained on 9,500 data points corresponding to Fig. S1(e-f).

Neural Network Model	Charges MAE (RMSE) [a.u.]
**Model 1**	0.026 (0.059)
**Model 2**	0.027 (0.060)
**Model 3**	0.026 (0.057)
**Model 4**	0.027 (0.057)
**Model 5**	0.027 (0.059)

To assess whether models can learn interaction energies, interaction forces, and atomic partial charges properly, we computed learning curves, which show the mean absolute error of several individually trained models for a given training set size in logarithmic scale. For proper learning, a linear decline is expected for the loss function on a log-log scale. The number of models that were trained for a given training set size was chosen such that the standard deviation of the MAE was within 1 kJ/mol (less than 10 meV).

The learning curves, in which each point shows the mean and the standard deviation of the mean absolute error (MAE) for 4-20 models trained on the interaction energy, interaction forces, and atomic partial charges, are shown in Figure S2, panels a, c, and e, respectively. The corresponding scatter plots of 5 models trained on interaction energies and interaction forces using the largest training set size are illustrated in panels b and d, respectively. For charges, only one model is used for dynamics, hence only one model is shown in panel f. The scatter plots are shown for the test set. In each plot, the systems for which the QM calculations are performed are indicated at the bottom. As can be seen from the scatter

plots, the model learns interaction energies and forces accurately. Except for a few data points with larger errors (that scatter stronger), model predictions almost perfectly match the reference values. Data points with larger errors are related to energetically unfavourable structures that were visited mainly during the last adaptive sampling run, mostly during dynamics with BuRNN on long time scales. As can be seen from the learning curves, all models learn properly, as a linear relation is observed when plotting the data on a log-log scale for the number of data points used for training and the MAEs of models. As is visible in panel f, the charge models scatter more strongly than the models for interaction energies and interaction forces. The learning curve shows that more data points could still improve the accuracy if needed. BuRNN simulations on long time scales were robust and led to smooth transitions in the forces between the buffer and outer regions (Figure S2), indicating that the accuracy of the charge predictions is sufficient for production runs.

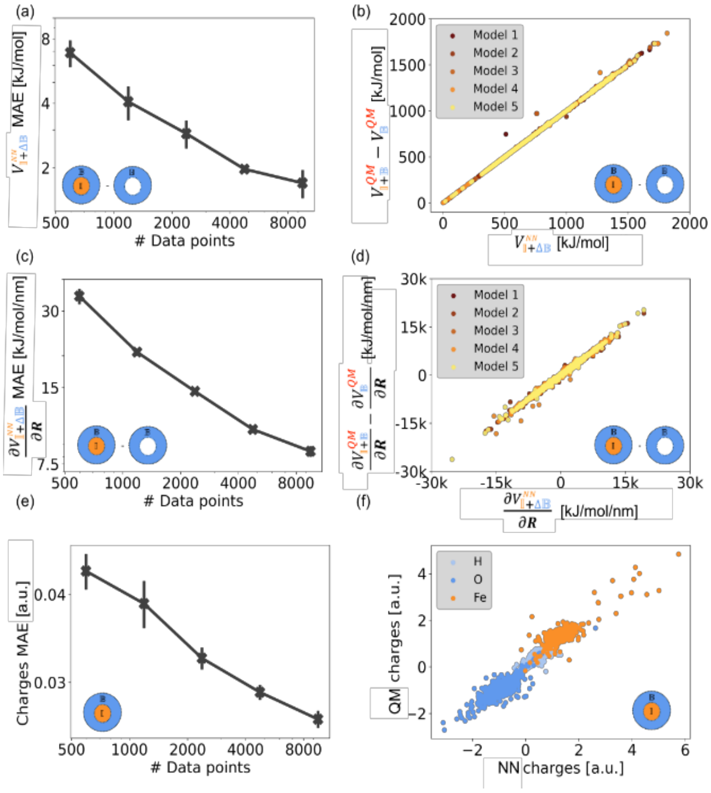

Figure S2: Learning curves for (a) interaction energies, (c) interaction forces, and (e) atomic partial charges that show the mean of 4-20 models trained on a given training set size including their standard deviation. Learning curves are shown in a logarithmic scale. Scatter plots for (b) interaction energies, (d) interaction forces, and (f) atomic partial charges of models trained on the largest training set size are shown. For adaptive sampling, at least two NN models are used for energies and forces, while only one model is used for atomic partial charges, hence only one model is shown. The interaction or region that is modelled is indicated at the bottom of each panel.

To investigate the performance of the model on larger inner regions, we additionally created a data set using a larger inner region with a cutoff of 2.5 nm from the central Fe^{3+} ion. We picked data points that were from SHAKEN simulations out of the original data set and 6211 calculations converged. We then trained neural networks on 5300 data points and used 300 and the rest for validation and testing, respectively. The errors we get when comparing two independently trained models using the smooth L1 loss function (section S1.3.1) are comparable to the models trained on similar amounts of data using an inner region that comprises the Fe^{3+} ion only.

The MAE (RMSE) for the models trained on the smaller inner region are 1.97 kJ/mol (4.73 kJ/mol) for energies and 12.1 kJ/mol/nm (42.3 kJ/mol/nm) MAE (RMSE) for forces. The models trained on the larger inner region have an MAE (RMSE) of 1.97 kJ/mol (7.9 kJ/mol) for energies and of 10.3 kJ/mol/nm (22.9 kJ/mol/nm) for forces, which is in both cases remarkably small.

These models were further used for BuRNN simulations. The radial distribution function is plotted for comparison in Figure S4 below. Results are comparable to those obtained from BuRNN with a smaller inner region.

S1.4 Molecular Dynamics Simulations

All molecular dynamics simulations were performed with a modified version of the GROMOS simulation package,[12, 13] with a direct interface to SchNetPack[10] modules using the pybind11 library.[14] As the code is deeply integrated into GROMOS, it will be part of the next release and will become freely available at www.gromos.net. Until then, the code is available upon request. Example input files to run a BuRNN simulation are provided.[15]

Simulations were performed in a periodic cubic box with box-edge lengths of 2.91 nm, containing one Fe^{3+} ion, and 786 SPC water molecules.[16] Temperature was maintained at 298 K using the Nosé Hoover chains scheme[17] by coupling the centre-of-mass motion and internal/rotational degrees of freedom to two separate temperature baths with coupling time of 0.1 ps and four chains. Bond lengths and angles in SPC water were constrained using the SHAKE algorithm.[18] Unless stated otherwise, a timestep of 2

fs was used. Lennard-Jones parameters of the Fe^{3+} atom were taken from the IOD parameter set of Li et al.[19] and the effective charge in classical simulations was set to +3.0. An additional set of simulations was performed with the 12-6-4 Lennard-Jones potential, in conjunction with the SPC/E water model, to account for the ion-induced dipole moment of the solvent, as derived by Li and Merz.[19] For the nonbonded MM interactions we applied a cutoff of 1.4 nm based on the distances between the heavy atoms (charge-group based cutoff) with a reaction-field of relative permittivity of 61 to account for a homogeneous medium outside the cutoff.[20] The pairlist was updated every step.

The BuRNN buffer region was created by applying a charge-group based cut-off of 0.5 nm from the central ion. Energies and forces were obtained from the previously trained Schnet models. We simultaneously used two models trained on the same data sets with different training and validation splits. The second model was used to validate the first model by comparing the energy predictions on-the-fly. Their mutual disagreement was used to pick snapshots for the next round of adaptive learning. Our implementation allows to monitor this quantity throughout the simulation. Partial atomic charges were obtained from another, adapted atomistic Schnet model[21] and applied to the Fe^{3+} ion and the water molecules inside the buffer region. To avoid double-counting of the water-water interactions within the buffer, their standard MM parameters and partial charges were used, as the polarization effects are added through the NN. The NN attributed charges were used only for the Coulombic interactions of the inner and buffer regions with the outer region. MM and BuRNN simulations were performed for 10 ns.

Simulations with a larger inner region, including the six coordinating waters were performed for 1 ns. As the current code does not allow for particles to change from the inner to the buffer region, additional half-harmonic attractive restraints were added to these waters. This ensures that the inner water molecules remain in the inner region and do not diffuse into the buffer or outer regions.

QM/MM simulations were performed using the central $Fe(H_2O)_6^{3+}$ complex as the QM region and the remaining water molecules as classical SPC water. QM calculations were performed by Gaussian 16, at the same functional and basis set as described above. Simulations were performed using both

mechanical and electrostatic embedding schemes, with nonpolar nonbonded interactions between the QM and MM regions described using the same force field parameters as described above. All water bonds and angles remained constrained using the SHAKE algorithm, allowing for a timestep of 2 fs and a total simulation time of 20 ps. In the mechanical embedding scheme, the coulombic interactions between the QM region and MM region were calculated using charges from the QM calculation every step. In the electrostatic embedding scheme, the MM charges up to 0.8 nm from the inner region were included in the SCF calculation.

The coordination number of the iron ion was determined using a 0.25 nm cutoff, based on an analysis of initial radial distribution functions. Hydrogen bonds were determined using a geometric criterion and were considered present if any hydrogen atom was within 0.25 nm of the oxygen atom of a neighbouring water molecule, while the O-H···O angle was at least 135°. Diffusion of the iron ion was determined using the Einstein equation by applying a linear fit to the mean square displacement over timescales of 0 – 0.5 ns.

S2 BuRNN simulations

S2.1 BuRNN forces

Figure S3 shows the profiles of the net forces on water molecules in panel a and their radial components in panel d. The net forces are smooth at the boundary (0.5 nm), which can be attributed to the fact that at the boundary the interaction with the inner region is small in comparison to the rest of the system. Contrary, the atom-wise contributions (panels c and f) exhibit jumps at the boundary, reflecting the transition from an electrostatic interaction based on localized point charges to a quantum mechanical interaction, mediated by delocalized electron densities. For complete molecules, these jumps disappear. Panels b and e show the contribution of the NN to the forces (either directly in $\boldsymbol{F}_{\mathbb{I}+\Delta\mathbb{B}}^{NN}$ or via NN-prediced partial charges in $\boldsymbol{F}_{\mathbb{B},\mathbb{O}}^{MM}$ and $\boldsymbol{F}_{\mathbb{I},\mathbb{O}}^{MM}$) is displayed. Within the buffer region, the forces reflect the observations in the radial distribution functions.

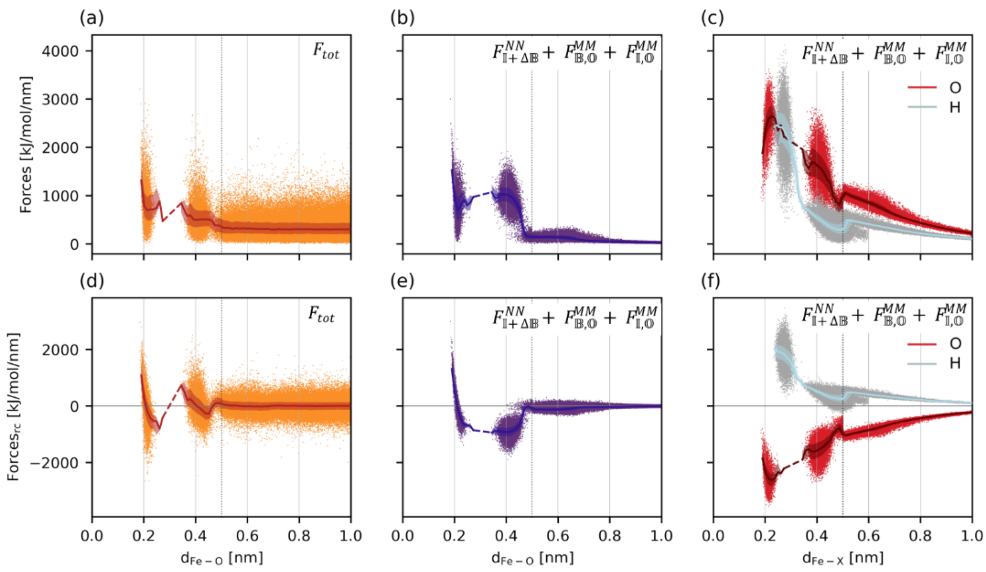

*Figure S3: Forces as a function of the distance to the iron atom (dots). The line represents the median and the band corresponds to the 2nd and 3rd quartile. Net forces on water molecules (**a**), contribution of the NN treatment of the inner and buffer region ($\boldsymbol{F}^{NN}_{\mathbb{I}+\Delta\mathbb{B}} + \boldsymbol{F}^{MM}_{\mathbb{B},\mathbb{O}} + \boldsymbol{F}^{MM}_{\mathbb{I},\mathbb{O}}$) to the net forces on the water molecules (**b**) and on the oxygen (red) and hydrogen (green) atoms (**c**). Panels **d**, **e** and **f** show radial components of the respective forces. Negative value means attraction.*

S2.2 Observed structure

Figure S4 shows the radial distribution functions for all simulations described in this work. As can be seen from the red and orange lines, BuRNN simulations with different inner regions are comparable. The orange curve is obtained from an inner region comprising the Fe^{3+} ion and the red curve is obtained from an inner region comprising the Fe^{3+} ion and the first solvation shell (a cutoff of 2.5 nm was chosen). Note that the peak corresponding to the second solvation shell resembles the one observed with

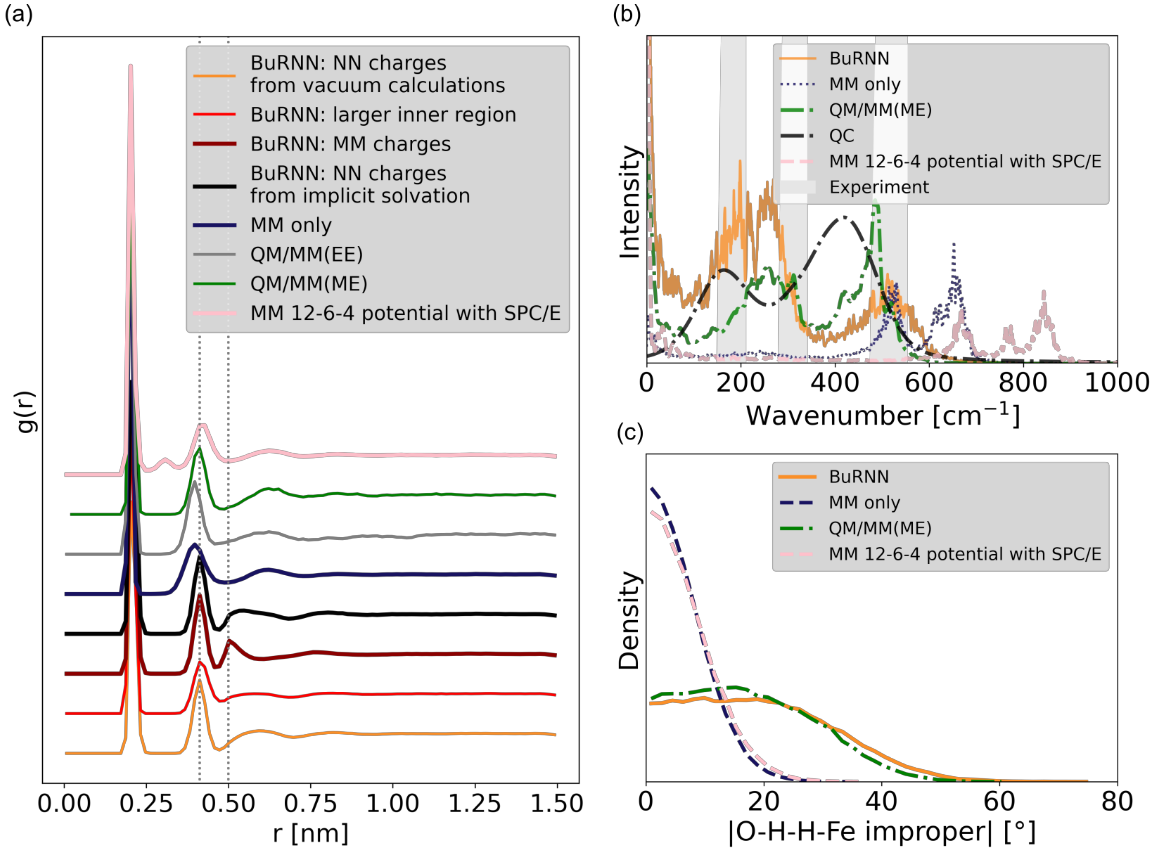

Figure S4: a) Iron-oxygen radial distribution function for different simulations settings. The blue curve shows the radial distribution function for a purely classical simulation (MM only), the orange (red) one for a regular BuRNN simulation (with a larger inner region), in which the partial charges for the inner and buffer region were determined from the QM calculations in vacuum. The black and dark red curves show the radial distribution function for a simulation in which partial charges for the inner and buffer region were estimated from QM calculations in an implicit solvent and assigned according to the classical force field, respectively. Grey and green lines show QM/MM simulations with density functional theory, once using an electrostatic embedding (EE) and once an mechanical embedding (EE), respectively. The pink line shows the radial distribution function obtained with MM 12-6-4 potential with SPC/E. Dotted lines indicated the second BuRNN peak and the cutoff used for the buffer region. Dashed lines indicate the second BuRNN peak and the cutoff used to define the buffer region b) Comparison of power spectra. QC refers to a quantum chemistry calculation of $Fe(H_2O)_{21}$ with the reference method used to generate the training set. Only modes related mostly to the Fe-O bond lengths are shown. The bands were created with a half-peak width of 100 cm^{-1} and for broadening a 1:1 mixture of Gaussian and Lorentzian functions were used. c) Probability distribution of the O-H-H-Fe improper dihedral angle obtained from BuRNN, MM only, QM/MM with EE and the MM 12-6-4 potential with SPC/E simulations.

QM/MM using mechanical embedding (abbreviated as ME in brackets), while for QM/MM with electrostatic embedding (abbreviated as EE in brackets) this peak is more similar to the MM only simulations. When using the 12-6-4 Lennard-Jones potential, an additional small peak between the first and second solvation shell is observed, corresponding to a water molecule that is in rapid exchange with the first shell. We have also performed simulations with different charge distributions on the inner and buffer regions in an overlay. Using partial charges from the force field for the inner and buffer regions leads to an artefact at the buffer region cutoff at 0.5 nm. Using (NN-predicted) charges from an additional implicit solvent QM calculation reduces, but does not remove this artefact. In these QM calculations we used the SMD variant of the integral-equation-formalism polarizable continuum model.[8] With the charges that are derived from gas-phase QM calculations, we obtain a smooth transition at 0.5 nm.

During the 10-ns BuRNN simulation the six coordinating water molecules did not exchange spontaneously. In the MM only description, a seventh water molecule very rarely moves within 0.25 nm of the central iron. In BuRNN, few individual water molecules occasionally vibrate beyond the 0.25 nm cutoff, leading to coordination numbers smaller than 6. No complete exchanges of the coordinating water molecules were observed in these simulations. In contrast, out of the total number of 786 water molecules, all visit the buffer region at least once during the 10-ns simulation. In total, 8,847 distinct visits to the buffer region were monitored (not counting the six coordinating water molecules), with an average lifetime of 14.4 ps. 8,579 visits had a lifetime of 5 ps or less; 268 visits to the buffer region had lifetimes longer than 5 ps, up to a maximum of 2,240 ps.

To assess differences in pure MM and BuRNN simulations, Figure 3 in the main text summarizes the geometries that are observed of the hexa-aqua iron complex. Figure S4 shows additional data for the simulations using QM/MM with mechanical embedding and for the classical simulations with the 12-6-4 Lennard-Jones potential. Note that consistently, the classical descriptions show an improper dihedral distribution that is centered around 0°, while the QM/MM and BuRNN simulations show wider distributions with an average value of 19 to 20°. While the distributions of distances are quite comparable in all simulations, the dynamics of the Fe-O bond is rather distinct. The power spectrum in

Figure 3d and Figure S4c was calculated as the Fourier transform of the autocorrelation function of the Fe-O bond lengths, as obtained in simulations in which these distances were recorded at every timestep for 100 ps (20 ps for the QM/MM simulations). Simulations using timesteps of 0.5 fs and of 2 fs are indistinguishable, with the period of the fastest vibrational motion around 50 to 60 fs, confirming that a timestep of 2 fs is suitable. The spectra obtained with BuRNN are most similar to those obtained from QM/MM calculations and are very different from both classical descriptions. In addition, we include the vibrational modes obtained from a quantum chemical calculation with the reference method using the optimized geometry and rigid-rotor/harmonic-oscillator approximation for $Fe(H_2O)_{21}$ in Figure S4c. Only modes related mostly to the Fe-O bond lengths are shown. The band is plotted as a superposition of broadened spectral lines of these modes using mixed Gaussian/Lorentzian functions (50:50) with a half-peak width of 100 cm^{-1} to resemble the shape of experimental spectra. These peaks are better aligned with BuRNN than with a pure MM simulation. The remaining differences between the QC spectrum and the observed frequencies in the power spectra can be explained by anharmonicities arising from the larger environment in the simulation. Moreover, the BuRNN spectra are in closer agreement with experimentally observed bands of 180 cm^{-1}, 310 cm^{-1} and 500 cm^{-1}.[22-24]

S2.3 BuRNN is robust with respect to higher temperatures

During BuRNN simulations at elevated temperatures, the disagreement between two NNs was monitored. The difference between the two NNs remained at an average value of –0.39 ± 0.02 kJ/mol with a standard deviation that increased from 1.17 kJ/mol at 300 K to 1.45 kJ/mol at 400 K, which is still very small and stays below chemical accuracy of 1 kcal/mol. Figure S5 shows the radial distribution functions and coordination numbers of 1-ns simulations at different temperatures. While the number of configurations in which a water molecule moves outside of the 0.25 nm cutoff increases, no complete exchanges of water molecules are observed at any of these temperatures.

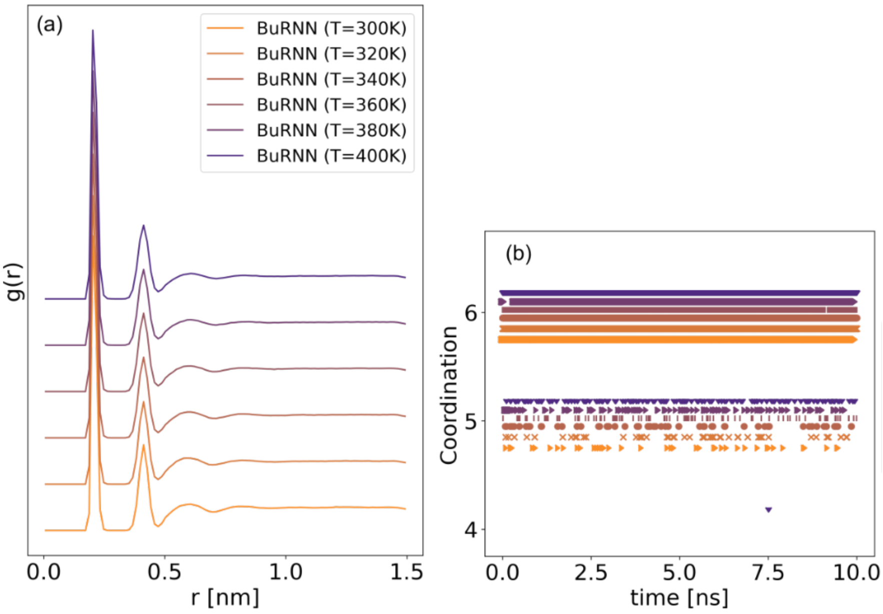

Figure S5: Iron-oxygen radial distribution functions for BuRNN simulations at different temperatures (left) and time series of the number of water molecules within 0.25 nm of the Fe3+ ion (with a small offset to visualize multiple simulations (right). Due to the size of the marker, the bars at a coordination of 6 seem continuous, but a small number of configurations is observed for which only 5 water molecules are within 0.25 nm.

Regarding simulations at which we pulled a water molecule away from Fe, we evaluated NN differences every 0.05 ps. The disagreement was found to be in the range of –0.68 ± 0.10 kJ/mol with a standard deviation of 1.47 kJ/mol, suggesting that the NNs are quite robustly sampling penta-coordinated iron complexes as well.

Movie S1: The file movie_S1.mp4 shows the spontaneous binding of a water molecule (cyan) to a pentacoordinated Fe^{3+}-water complex. The configuration switches from a trigonal bipyramidal arrangement to an octahedral complex in the process. Fe^{3+} as orange sphere, coordinating waters in sticks, buffer waters in blue, water in the outer region in grey.

References

(1) Frisch, M. J.; Trucks, G. W.; Schlegel, H. B.; Scuseria, G. E.; Robb, M. A.; Cheeseman, J. R.; Scalmani, G.; Barone, V.; Petersson, G. A.; Nakatsuji, H.; Li, X.; Caricato, M.; Marenich, A. V.; Bloino, J.; Janesko, B. G.; Gomperts, R.; Mennucci, B.; Hratchian, H. P.; Ortiz, J. V.; Izmaylov, A. F.; Sonnenberg, J. L.; Williams; Ding, F.; Lipparini, F.; Egidi, F.; Goings, J.; Peng, B.; Petrone, A.; Henderson, T.; Ranasinghe, D.; Zakrzewski, V. G.; Gao, J.; Rega, N.; Zheng, G.; Liang, W.; Hada, M.; Ehara, M.; Toyota, K.; Fukuda, R.; Hasegawa, J.; Ishida, M.; Nakajima, T.; Honda, Y.; Kitao, O.; Nakai, H.; Vreven, T.; Throssell, K.; Montgomery Jr., J. A.; Peralta, J. E.; Ogliaro, F.; Bearpark, M. J.; Heyd, J. J.;

Brothers, E. N.; Kudin, K. N.; Staroverov, V. N.; Keith, T. A.; Kobayashi, R.; Normand, J.; Raghavachari, K.; Rendell, A. P.; Burant, J. C.; Iyengar, S. S.; Tomasi, J.; Cossi, M.; Millam, J. M.; Klene, M.; Adamo, C.; Cammi, R.; Ochterski, J. W.; Martin, R. L.; Morokuma, K.; Farkas, O.; Foresman, J. B.; Fox, D. J. *Gaussian 16 rev. C.01*, Wallingford, CT, 2016.

(2) Radoń, M., Benchmarking quantum chemistry methods for spin-state energetics of iron complexes against quantitative experimental data. *Phys. Chem. Chem. Phys.* **2019,** *21*, 4854-4870.

(3) Hoe, W.-M.; Cohen, A. J.; Handy, N. C., Assessment of a new local exchange functional optx. *Chem. Phys. Lett.* **2001,** *341*, 319-328.

(4) Perdew, J. P.; Burke, K.; Ernzerhof, M., Generalized gradient approximation made simple. *Phys. Rev. Lett.* **1996,** *77*, 3865-3868.

(5) Weigend, F.; Ahlrichs, R., Balanced basis sets of split valence, triple zeta valence and quadruple zeta valence quality for h to rn: Design and assessment of accuracy. *Phys. Chem. Chem. Phys.* **2005,** *7*, 3297-3305.

(6) Singh, U. C.; Kollman, P. A., An approach to computing electrostatic charges for molecules. *J. Comput. Chem.* **1984,** *5*, 129-145.

(7) Besler, B. H.; Merz Jr., K. M.; Kollman, P. A., Atomic charges derived from semiempirical methods. *J. Comput. Chem.* **1990,** *11*, 431-439.

(8) Marenich, A. V.; Cramer, C. J.; Truhlar, D. G., Universal solvation model based on solute electron density and on a continuum model of the solvent defined by the bulk dielectric constant and atomic surface tensions. *J. Phys. Chem. B* **2009,** *113*, 6378-6396.

(9) Schütt, K. T.; Sauceda, H. E.; Kindermans, P.-J.; Tkatchenko, A.; Müller, K.-R., Schnet – a deep learning architecture for molecules and materials. *J. Chem. Phys.* **2018,** *148*, 241722.

(10) Schütt, K. T.; Kessel, P.; Gastegger, M.; Nicoli, K. A.; Tkatchenko, A.; Müller, K. R., Schnetpack: A deep learning toolbox for atomistic systems. *J. Chem. Theory Comput.* **2019,** *15*, 448-455.

(11) Gastegger, M.; Behler, J.; Marquetand, P., Machine learning molecular dynamics for the simulation of infrared spectra. *Chem. Sci.* **2017,** *8*, 6924-6935.

(12) Eichenberger, A. P.; Allison, J. R.; Dolenc, J.; Geerke, D. P.; Horta, B. A. C.; Meier, K.; Oostenbrink, C.; Schmid, N.; Steiner, D.; Wang, D.; van Gunsteren, W. F., Gromos++ software for the analysis of biomolecular simulation trajectories. *J. Chem. Theory Comput.* **2011,** *7*, 3379-3390.

(13) Schmid, N.; Christ, C. D.; Christen, M.; Eichenberger, A. P.; van Gunsteren, W. F., Architecture, implementation and parallelisation of the gromos software for biomolecular simulation. *Comput. Phys. Commun.* **2012,** *183*, 890-903.

(14) Wenzel, J.; Rhinelander, J.; Moldovan, D. Pybind11 - seamless operability between c++11 and python. https://rgl.epfl.ch/software/pybind11.

(15) Westermayr, J.; Lier, B.; Poliak, P.; Marquetand, P.; Oostenbrink, C., Burnn. figshare: 2021.

(16) Berendsen, H. J. C.; Postma, J. P. M.; van Gunsteren, W. F.; Hermans, J., Interaction models for water in relation to protein hydration. In *Intermolecular forces: Proceedings of the fourteenth jerusalem symposium on quantum chemistry and biochemistry held in jerusalem, israel, april 13–16, 1981*, Pullman, B., Ed. Springer Netherlands: Dordrecht, 1981; pp 331-342.

(17) Hoover, W. G., Canonical dynamics: Equilibrium phase-space distributions. *Phys. Rev. A* **1985,** *31*, 1695-1697.

(18) Ryckaert, J.-P.; Ciccotti, G.; Berendsen, H. J. C., Numerical integration of the cartesian equations of motion of a system with constraints: Molecular dynamics of n-alkanes. *J. Comput. Phys.* **1977,** *23*, 327-341.

(19) Li, P.; Song, L. F.; Merz, K. M., Parameterization of highly charged metal ions using the 12-6-4 lj-type nonbonded model in explicit water. *J. Phys. Chem. B* **2015,** *119*, 883-895.

(20) Tironi, I. G.; Sperb, R.; Smith, P. E.; van Gunsteren, W. F., A generalized reaction field method for molecular dynamics simulations. *J. Chem. Phys.* **1995,** *102*, 5451-5459.

(21) SchNetPack *Https://github.Com/juliawestermayr/schnetpack*, 2022.

(22) Ohtaki, H.; Radnai, T., Structure and dynamics of hydrated ions. *Chem. Rev.* **1993,** *93*, 1157-1204.

(23) Böhm, F.; Sharma, V.; Schwaab, G.; Havenith, M., The low frequency modes of solvated ions and ion pairs in aqueous electrolyte solutions: Iron(ii) and iron(iii) chloride. *Phys. Chem. Chem. Phys.* **2015,** *17*, 19582-19591.
(24) Mink, J.; Németh, C.; Hajba, L.; Sandström, M.; Goggin, P. L., Infrared and raman spectroscopic and theoretical studies of hexaaqua metal ions in aqueous solution. *J. Mol. Struc.* **2003,** *661-662*, 141-151.
