# Supplementary material for: BuRNN: Buffer Region Neural Network Approach for Polarizable-Embedding Neural Network/Molecular Mechanics Simulations
Source: J Phys Chem Lett. 2022 Apr 25;13(17):3812–8. doi: 10.1021/acs.jpclett.2c00654 (PMC9082612; doi:10.1021/acs.jpclett.2c00654)
Supplement: Supplementary file 3 — jz2c00654_si_003.pdf [file jz2c00654_si_003.pdf]

jz-2022-00654k.R1

Name: Peer Review Information for "BuRNN: Buffer Region Neural Network Approach for Polarizable-Embedding Neural Network / Molecular Mechanics Simulations"

First Round of Reviewer Comments

Reviewer: 1

Comments to the Author

The authors introduce a new method for hybrid quantum-classical simulations in the condensed phase. They use a neural network to learn the energy of a quantum subsystem and the quantum polarization of a classical buffer region. Interactions within the buffer, and between the outer region and all other regions, are treated with MM. The work is novel and creative, addressing both interface issues and computational cost in QM/MM. The agreement with experiment is impressive, especially compared to the results of pure MM. I highly recommend this article for publication in JPC letters, after addressing the following comments.

Suggestions:

- I found the description of how BuRNN works, starting with Equation (1), to be confusing. In particular, I found it difficult to understand why certain coupling terms were showing up at different levels of theory and how they would help alleviate the QM/MM boundary problems. I understood it after reading it over several times, but many readers might give up after the first try. To increase the paper's readability, and hence its impact, I recommend the following:

o Provide a motivation for the term ($V_B^{MM} - V_B^{QM}$) before writing down Eq. (1). As I understand it, writing $V_{tot} = V_{I + B}^{QM} + V_{I(I + B + O)}$ would be the same as regular QM/MM with (I + B) given by QM, and hence would have boundary problems. So ($V_B^{MM} - V_B^{QM}$) helps to smooth the transition between the regions. While that's explained in more depth during the discussion around Eqs. (2)-(6), I think saying it earlier on would make things much clearer.

o To further clarify the discussion around Eq. (1), I would split up the sentence before Eq. (1) into multiple shorter sentences. I found it difficult to understand this sentence because it was so long, and because so many variables were introduced. Also, related to the above point, the sentence tells you what each term in Eq. (1) represents, but not why it's there. I might say something like this instead: "The total potential energy contains the QM energy of the inner and buffer regions, and the MM energy coupling the outer region to all others. It additionally contains $V_B^{MM} - V_B^{QM}$, the difference between the MM buffer energy and the QM buffer energy. This term helps to smooth the transition between QM in the inner region and MM in the outer region; this is discussed in further detail below. Adding these terms together, we get Eq. (1)".

o I would get rid of the bracket notation in terms like $V^{QM}_{B(I+B), B(I+B)}$. To me they didn't clearly express the concept of a pairwise interaction in the context of an IB calculation. Further, their use is inconsistent: if the same notation were used throughout, then $V_{I, I}^{QM}$ would be replaced with $V_{I(I+B), I(I+B)}^{QM}$. I understand why the authors didn't write this, because there was never an isolated $V_{I, I}$ to compare to, but this inconsistency made me even more confused.

It might be clearer to write the following:

"$V_{I + B}^{QM} = V_{I, I}^{QM} + V_{I, B}^{QM} + V_{B, B}^{QM}$ (2)

$V_{\Delta B}^{QMpol} = V{B, B}^{QM} – V_{B, B}^{QM, isolated}$ (4) [delete Eq. (3)].

Each term comes from a full I + B calculation, except for $V_{B, B}^{QM, isolated}$, which comes from an isolated B calculation."

To me, the new Eq. (4) clearly expresses the meaning of $V_{\Delta B}^{QMpol}$ and how it is calculated. It's true that the fact that $V_{I, I}^{QM}$ and $V_{B, B}^{QM}$ are computed in the context of an I + B calculation is not as explicit as it was originally. However, I think that it comes across clearly since Eq. (4) is now right below Eq. (2). Further, the notation is more consistent now, since we are not giving the (I+B) label to $V_{B, B}$ without also giving it to $V_{I, I}$.

o I would clarify the difference in meaning between the bracket notation in Eqs. (1, 5) and Eq. (2). In Eqs. (1) and (5), $V_{O(I+B+O)}$ seems to mean the interaction between O and the regions I, B, and O, which is not the same meaning as "V_O in the context of an I + B + O QM calculation".

o I also don't feel that the notation $V_{\Delta B}^{QMpol}$ is intuitive. If Delta is supposed to reflect a change, which is due to the polarization from the QM region I, then why is QMpol also in the superscript? Wouldn't Delta and QMpol mean the same thing? Also, this term is supposed to mean the change in B, B interaction due to polarization from I, right? If that's the case, then I think it would be better to just write $V_{B, B}^{QMpol}$, to make the notation consistent with all the other pairwise terms in Eq. (6).

o I think another key point in Eqs. (1) and (6) is that the I-induced polarization in B modifies the partial charges of B, and these different charges couple to O. While this is explained in the second paragraph after Eq. (6), I feel it would be helpful to mention it around Eq. (1) as well.

o Can you explain this sentence in more depth? "One of the benefits is that any artefacts in the electronic degrees of freedom due to the interface to the outer region will largely cancel in the difference $V_{I+B}^{QM} – V_{B}^{QM}$, assuming that the relevant polarization of the buffer region predominantly takes place at the interface between the inner and the buffer region." What is an example of an artifact that you might see at the interface, and why does it cancel in the difference?

- I would also cite FieldSchNet as an example of combining QM with MM [M. Gastegger, K. T. Schutt, and K-R Muller, 2021. Chemical Science, 12(34), pp. 11473-11483]

- Is the idea of a buffer region something new that was introduced in this work, or has it been proposed before? If it is new then I would emphasize this point, to further drive home the novelty of the

approach. If it is not new, then I would clarify that it has been proposed before, but that it was computationally expensive without a neural network.

- The accuracy of the model is outstanding – I would emphasize this more in the main text. Right now the MAEs are simply quoted, but the reader doesn't necessarily understand how good they are

- In the discussion of the O-H-H-Fe improper dihedral angle, I would also report the mean angle predicted by BuRNN so it can be compared to the BP86/COSMO prediction.

Reviewer: 2

Comments to the Author

The manuscript by Lier et al. describes the development of a ML/MM framework for molecular dynamics simulations. The approach is based on the partitioning of a system between inner, buffer, and outer regions, where the inner/buffer interaction is learned from QM calculation through a neural network, whereas the buffer/outer interaction is described at the MM level. The training of this model requires two QM calculations to obtain the inner/buffer interaction energy. The advantage of this approach is that all the interactions within the inner and buffer regions are in principle of QM quality. On the other hand, the interaction between buffer and outer region is computed at the MM level. The approach is then applied to the description of Fe(III) in water.

This contribution is interesting in that it pushes the boundary of machine learning methods to a multiscale description of the potential energy. However, the method is applied on a rather simple system and compared to a very crude MM potential. Moreover, the method has not been validated against a QM/MM simulation. This approach is surely in principle interesting, but there is no sufficient proof as regards its general applicability. I think that a more specialized computational chemistry journal would be more appropriate for this manuscript.

Major points:

1) The authors claim to have "validated" their method, but all they did was a comparison with a full MM model and experimental data. They find differences especially in the water orientation with respect to the Fe ion, and in the power spectrum of the Fe-O bond. This is not really surprising, given that the MM model only describes the Fe/water interaction with a Coulomb plus Lennard-Jones potential. A more sensible comparison should be done with a QM/MM model where the QM part is the Fe ion plus the first water solvation shell. This model would be enough to properly compare the quantities shown in Figure 3.

2) The BuRNN MD is compared with a MM MD with a very approximate water model (SPC). In addition, no corrections were considered for the ion-induced dipole interactions, such as through a 1/R^4 term added to the standard Lennard-Jones potential [J. Chem. Theory Comput., 2014, 10, 289-297]. The 12-6-4 model was parametrized for trivalent ions and several water models [J. Phys. Chem. B, 2015, 119, 883-895], therefore it should be readily applicable to the current system. This is still a pure MM description,

with negligible additional cost compared to standard MM potential (i.e. less expensive than a ML model). As such, it would be a more sensible comparison for the BuRNN approach.

3) The application presented here is a very special case in which only one atom is included in the inner region. This obviously simplifies the problem by reducing (i) the number of features needed for learning and (ii) the variance of the target quantities. I wonder whether this approach is really scalable to e.g. a solute molecule in water, or any coordination compound with different ligands.

4) The largest difference between BuRNN and MM is in the power spectrum shown in Figure 3. This is compared to experimental FTIR spectra of FeCl3. However, while the FTIR spectra reveal clearly separated bands, the power spectra show a very noisy band around 200 cm-1. In addition, experimental spectra of ref. 47 also contain the effect of Cl ions in the coordination shell, which changes both the symmetry and the reduced mass of the oscillator. I am not sure that the comparison with experiments is clean enough to be used as a validation of the model.

Other points:

5) If I understood correctly, ESP atomic charges are calculated at the QM level on the inner+buffer region. Since this region is quite large, the ESP charges of atoms far from the surface are probably not well fitted. Have the authors checked if the ESP charges of "internal" waters are similar to those of "surface" waters?

6) In the SI it is stated that the Fe3+ LJ parameters were taken from Zou et al., but in that reference it seems that LJ parameters were derived for Fe in the Heme group. I wonder if this can bias the MM level towards a worse agreement with experiments.

7) Figure S3 compares the RDFs calculated with different definitions for the charges (MM vs NN-implicit vs NN-vacuum). The authors claim that there is an artifact when using MM charges, but it is difficult to notice it from Figure S3, especially since the relevant RDFs are represented with dashed lines.

8) The training protocol used here seems to be a very ad-hoc and cumbersome procedure. There is a step in which 21 configurations from a preliminary BurNN MD were extracted and minimized at the QM level, then 4000 configurations are extracted from the minimization trajectories (is this correct?). Then, 4000 (additional?) configurations are obtained by applying SHAKE and removing the water molecules which moved outside the cutoff. Finally, in addition to the adaptive training, ad-hoc configurations with a lower coordination number were generated. I wonder how much this training process is really applicable to more complex systems.

Author's Response to Peer Review Comments:

Department of Material Sciences and Process Engineering
Institute for Molecular Modeling and Simulation

Univ. Prof. Dr. Chris Oostenbrink

Manuscript: **jz-2022-00654k**
Title: **BuRNN: Buffer Region Neural Network Approach for polarizable-embedding NN/MM Simulations**
Authors: **Bettina Lier, Peter Poliak, Philipp Marquetand, Julia Westermayr, Chris Oostenbrink**

Vienna, 14.4.2022.

Dear Professor Editor,

Thank you for your e-mail of March 22nd, concerning the above-mentioned manuscript. We have carefully read your editorial comments and the concerns of the reviewers. We thank both reviewers for their constructive feedback. We have performed a number of additional simulations and analyses and have considerably revised the manuscript, which we are now ready to submit. We also submit a version of the manuscript in which all changes are highlighted.

Let me respond to the various points one-by-one, using blue color to distinguish our answers from the comments of the reviewers:

Editorial comments

1. Title: In both the main manuscript file and the Supporting Information, set the title in title case, with the first letter of each principal word capitalized.

We have made this modification

2. Title: Using acronyms in title is discouraged. Please spell out all acronyms in the title of the manuscript and Supporting Information.

We have spelled out the abbreviation for Neural Network / Molecular Mechanics simulations. We suggest to keep the BuRNN acronym, as the title basically introduces this abbreviation.

3. TOC Graphic: Please resize per journal guidelines (2 in x 2 in).

We have modified the TOC graphic according to your instructions.

4. Supporting Information: Please add full header at top of page of the Supporting Information file, which includes: Title, Full Author List, and Author affiliations (exactly as they appear in the manuscript).

We have modified the Supporting Information accordingly.

5. Title: Title must match in three places: (1) manuscript file, (2) supporting information, and (3) ACS Paragon Plus.

We apologize for the mismatches and have made sure that the title is now consistent throughout.

6. Supporting Information Statement: A brief, nonsentence description of the actual contents of each supporting information file is required. This description should be labeled Supporting Information and should appear before the Acknowledgement and Reference sections.

We have added this statement to the revised manuscript.

*7. References: In both the main file and the supporting information, fix the style of all references to use JPCL formatting (check all references carefully). ***JPC Letters reference formatting requires that journal references should contain: () around numbers, author names, article title (titles entirely in title case or entirely in lower case), abbreviated journal title (italicized), year (bolded), volume (italicized), and pages (first-last). Book references should contain author names, book title (in the same pattern), publisher, city, and year.*

We apologize that the references were not yet in order and have fixed the ones that did not match the proper style in the revised manuscript.

Reviewer 1

Recommendation: This paper is probably publishable, but major revision is needed; I do not need to see future revisions.

Comments:

The authors introduce a new method for hybrid quantum-classical simulations in the condensed phase. They use a neural network to learn the energy of a quantum subsystem and the quantum polarization of a classical buffer region. Interactions within the buffer, and between the outer region and all other regions, are treated with MM. The work is novel and creative, addressing both interface issues and computational cost in QM/MM. The agreement with experiment is impressive,

especially compared to the results of pure MM. I highly recommend this article for publication in JPC letters, after addressing the following comments.

We thank the reviewer for the positive evaluation of our work. We have tried to accommodate the remaining concerns as outlined below.

Suggestions:

- I found the description of how BuRNN works, starting with Equation (1), to be confusing. In particular, I found it difficult to understand why certain coupling terms were showing up at different levels of theory and how they would help alleviate the QM/MM boundary problems. I understood it after reading it over several times, but many readers might give up after the first try. To increase the paper's readability, and hence its impact, I recommend the following:

o Provide a motivation for the term ($V_B^{MM} – V_B^{QM}$) before writing down Eq. (1). As I understand it, writing $V_{tot} = V_{I + B}^{QM} + V_{I(I + B + O)}$ would be the same as regular QM/MM with (I + B) given by QM, and hence would have boundary problems. So ($V_B^{MM} – V_B^{QM}$) helps to smooth the transition between the regions. While that's explained in more depth during the discussion around Eqs. (2)-(6), I think saying it earlier on would make things much clearer.

We thank the reviewer for this suggestion. We have revised the text surrounding equation 1 to clarify the motivation of these terms earlier on.

o To further clarify the discussion around Eq. (1), I would split up the sentence before Eq. (1) into multiple shorter sentences. I found it difficult to understand this sentence because it was so long, and because so many variables were introduced. Also, related to the above point, the sentence tells you what each term in Eq. (1) represents, but not why it's there. I might say something like this instead: "The total potential energy contains the QM energy of the inner and buffer regions, and the MM energy coupling the outer region to all others. It additionally contains $V_B^{MM} – V_B^{QM}$, the difference between the MM buffer energy and the QM buffer energy. This term helps to smooth the transition between QM in the inner region and MM in the outer region; this is discussed in further detail below. Adding these terms together, we get Eq. (1)".

We agree with the reviewer that shortening sentences often helps to clarify the meaning. The indicated sentence was modified in the context of the previous comment and we have tried to be clearer in our description of the terms.

o I would get rid of the bracket notation in terms like $V^{QM}_{B(I+B), B(I+B)}$. To me they didn't clearly express the concept of a pairwise interaction in the context of an IB calculation. Further,

their use is inconsistent: if the same notation were used throughout, then V_{I, I}^{QM} would be replaced with V_{I(I+B), I(I+B)}^{QM}. I understand why the authors didn't write this, because there was never an isolated V_{I, I} to compare to, but this inconsistency made me even more confused.

It might be clearer to write the following:

"V_{I + B}^{QM} = V_{I, I}^{QM} + V_{I, B}^{QM} + V_{B, B}^{QM} (2)

V_{\Delta B}^{QMpol} = V{B, B}^{QM} – V_{B, B}^{QM, isolated} (4) [delete Eq. (3)].

Each term comes from a full I + B calculation, except for V_{B, B}^{QM, isolated}, which comes from an isolated B calculation."

To me, the new Eq. (4) clearly expresses the meaning of V_{\Delta B}^{QMpol} and how it is calculated. It's true that the fact that V_{I, I}^{QM} and V_{B, B}^{QM} are computed in the context of an I + B calculation is not as explicit as it was originally. However, I think that it comes across clearly since Eq. (4) is now right below Eq. (2). Further, the notation is more consistent now, since we are not giving the (I+B) label to V_{B, B} without also giving it to V_{I, I}.

We thank the reviewer for this suggestion and we agree that the less formal way of writing it with the indication 'isolated' improves the clarity of the text. We have adopted the suggestion in the revised manuscript.

o I would clarify the difference in meaning between the bracket notation in Eqs. (1, 5) and Eq. (2). In Eqs. (1) and (5), V_{O(I+B+O)} seems to mean the interaction between O and the regions I, B, and O, which is not the same meaning as "V_O in the context of an I + B + O QM calculation".

We agree with the reviewer that this is not the same meaning as the brackets we had before. In response of the previous point, we have removed the bracket notation for 'in the context of', and therefore think that we should keep the notation O(I+B+O) here, to indicate that this term contains all interactions with the outer region.

o I also don't feel that the notation V_{\Delta B}^{QMpol} is intuitive. If Delta is supposed to reflect a change, which is due to the polarization from the QM region I, then why is QMpol also in the superscript? Wouldn't Delta and QMpol mean the same thing? Also, this term is supposed to mean the change in B, B interaction due to polarization from I, right? If that's the case, then I think it would be better to just write V_{B, B}^{QMpol}, to make the notation consistent with all the other pairwise terms in Eq. (6).

We had added the Delta term to indicate that this term essentially reflects a delta-learning, where the neural network learns the difference between a MM and a QM description for the buffer region. We understand that this term confused the reviewer and we have now returned to the notation V_{BB}^{QMpol} as suggested by the reviewer.

o I think another key point in Eqs. (1) and (6) is that the I-induced polarization in B modifies the partial charges of B, and these different charges couple to O. While this is explained in the second paragraph after Eq. (6), I feel it would be helpful to mention it around Eq. (1) as well.

We agree with the reviewer that this is also a key point of the method. Unfortunately, we cannot say everything 'in the beginning'. In our rewrite of the text around these equations we have tried to emphasize the relevance of this effect more. We now first mention this point just above equation 4.

o Can you explain this sentence in more depth? "One of the benefits is that any artefacts in the electronic degrees of freedom due to the interface to the outer region will largely cancel in the difference V_{I+B}^{QM} – V_{B}^{QM}, assuming that the relevant polarization of the buffer region predominantly takes place at the interface between the inner and the buffer region." What is an example of an artifact that you might see at the interface, and why does it cancel in the difference?

We thank the reviewer for pointing out that this was not clear enough as it is another essential feature of the BuRNN approach. If one does a QM calculation in vacuum, the electron density at the vacuum boundary is likely different than if there would be a continuous aqueous phase. In the BuRNN approach, the corresponding error we make in the QM calculations is first of all relatively far from the inner region, but additionally, we make a similar error twice and its effect will largely cancel in the difference $V_{I+B}^{QM} – V_{B}^{QM}$. We have clarified this in the revised manuscript.

- I would also cite FieldSchNet as an example of combining QM with MM [M. Gastegger, K. T. Schutt, and K-R Muller, 2021. Chemical Science, 12(34), pp. 11473-11483] - Is the idea of a buffer region something new that was introduced in this work, or has it been proposed before? If it is new then I would emphasize this point, to further drive home the novelty of the approach. If it is not new, then I would clarify that it has been proposed before, but that it was computationally expensive without a neural network.

We apologize that we did not explicitly mention FieldSchNet, which had fallen out of the original manuscript. We now mention it explicitly in the revised manuscript. Other approaches with a buffer region have been suggested in the past and schemes like the ONIOM approach also define different regions, but to the best of our knowledge the exact buffered approach as described here has not been used before. We emphasize this in the revised manuscript.

- The accuracy of the model is outstanding – I would emphasize this more in the main text. Right now the MAEs are simply quoted, but the reader doesn't necessarily understand how good they are

We thank the reviewer for pointing the accuracy of our NN models out. We have emphasized the accuracy of the models more in the revised manuscript on page 4 and 9. We also mentioned that the high accuracy can be achieved because interaction energies are trained instead of potential energies, which span a smaller energy range.

- In the discussion of the O-H-H-Fe improper dihedral angle, I would also report the mean angle predicted by BuRNN so it can be compared to the BP86/COSMO prediction.

Another good suggestion by the reviewer. We have added the average value of 19.3 ° to the results section. This value agrees nicely with the optimal value of 16 °, and also with the average as observed in newly performed QM/MM simulations of 20.3 °. This is now all mentioned in the text.

Additional Questions:

Urgency: High

Significance: Top 10%

Novelty: Top 10%

Scholarly Presentation: Moderate

Is the paper likely to interest a substantial number of physical chemists, not just specialists working in the authors' area of research?: Yes

Reviewer: 2

Recommendation: While the work is good and publishable, a more appropriate journal is recommended such as JCTC

We thank the reviewer for the general positive evaluation of our work. We still think that the novel method is of broader interest than the readers of the Journal of Chemical Theory and Computation. The development of a novel approach to mix different levels of theory is not just of interest of

computational chemistry community, but also of interest to the broader physical chemistry area, as it opens the way to more accurate models.

Comments:

The manuscript by Lier et al. describes the development of a ML/MM framework for molecular dynamics simulations. The approach is based on the partitioning of a system between inner, buffer, and outer regions, where the inner/buffer interaction is learned from QM calculation through a neural network, whereas the buffer/outer interaction is described at the MM level. The training of this model requires two QM calculations to obtain the inner/buffer interaction energy. The advantage of this approach is that all the interactions within the inner and buffer regions are in principle of QM quality. On the other hand, the interaction between buffer and outer region is computed at the MM level. The approach is then applied to the description of Fe(III) in water.

This contribution is interesting in that it pushes the boundary of machine learning methods to a multiscale description of the potential energy. However, the method is applied on a rather simple system and compared to a very crude MM potential. Moreover, the method has not been validated against a QM/MM simulation. This approach is surely in principle interesting, but there is no sufficient proof as regards its general applicability. I think that a more specialized computational chemistry journal would be more appropriate for this manuscript.

Major points:

1) The authors claim to have "validated" their method, but all they did was a comparison with a full MM model and experimental data. They find differences especially in the water orientation with respect to the Fe ion, and in the power spectrum of the Fe-O bond. This is not really surprising, given that the MM model only describes the Fe/water interaction with a Coulomb plus Lennard-Jones potential. A more sensible comparison should be done with a QM/MM model where the QM part is the Fe ion plus the first water solvation shell. This model would be enough to properly compare the quantities shown in Figure 3.

We thank the reviewer for this comment, which we found refreshing as we (as computational chemists) are often asked to compare to experiment rather than theory. It was thus our pleasure to follow up on this recommendation and to compare our BuRNN approach to conventional QM/MM simulations, both with mechanical embedding and with electrostatic embedding. As in the initial version, we compare the radial distribution functions, bond lengths, improper dihedral angle, and power spectra obtained with the different methods in Fig. 3 and Fig. S4 (previous Fig. S3) and reproduce Fig. 3 here which shows results for the QM/MM simulation with electrostatic embedding (EE). Results using mechanical embedding are very similar and plotted in Fig. S4 (previous S3). The data show that the BuRNN simulations agree closely to the QM/MM simulations, in particular

for the improper dihedral angle and the power spectra, where the differences between BuRNN and a classical approach were largest.

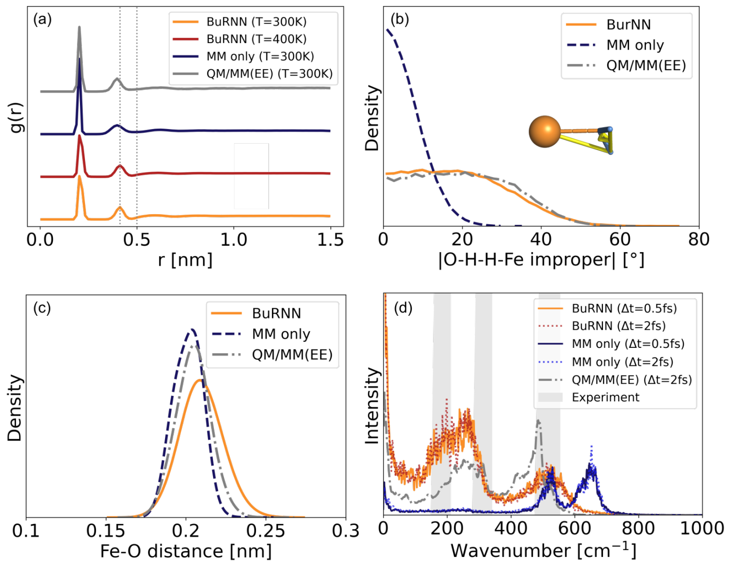

Figure 2: Coordination of Fe3+ by water molecules with BuRNN simulations and when using MM only. a) radial distribution function for BuRNN at a temperature of 300K and 400K, with MM only and a QM/MM simulation using electrostatic embedding (EE); dashed lines indicate the second BuRNN peak and the cutoff used to define the buffer region. b) Probability distribution of the O-H-H-Fe improper dihedral, c) distribution of the Fe-O distance, and d) power spectrum of the Fe-O coordinative bond for different simulations. Experimental data are taken from Refs. 50-52 [see manuscript].

2) The BuRNN MD is compared with a MM MD with a very approximate water model (SPC). In addition, no corrections were considered for the ion-induced dipole interactions, such as through a 1/R^4 term added to the standard Lennard-Jones potential [J. Chem. Theory Comput., 2014, 10, 289-297]. The 12-6-4 model was parametrized for trivalent ions and several water models [J. Phys. Chem. B, 2015, 119, 883-895], therefore it should be readily applicable to the current system. This is still a pure MM description, with negligible additional cost compared to standard MM potential (i.e. less expensive than a ML model). As such, it would be a more sensible comparison for the BuRNN approach.

Another very useful suggestion by the reviewer. We have performed simulations of the Fe^{3+} ion with the 12-6-4 Lennard-Jones potential, in conjunction with SPC/E water (as it was parameterized in the work of Li et al.). We find that while this parameter set maintains the hexa-coordinated structure very nicely, an additional peak in the radial distribution function appears. Furthermore, the Fe^{3+} - H_2O interactions appear to be very tight, with even higher frequencies in the power

spectrum. We have included this data in figure S4 (previous figure S3) of the supporting information and reproduce the figure below.

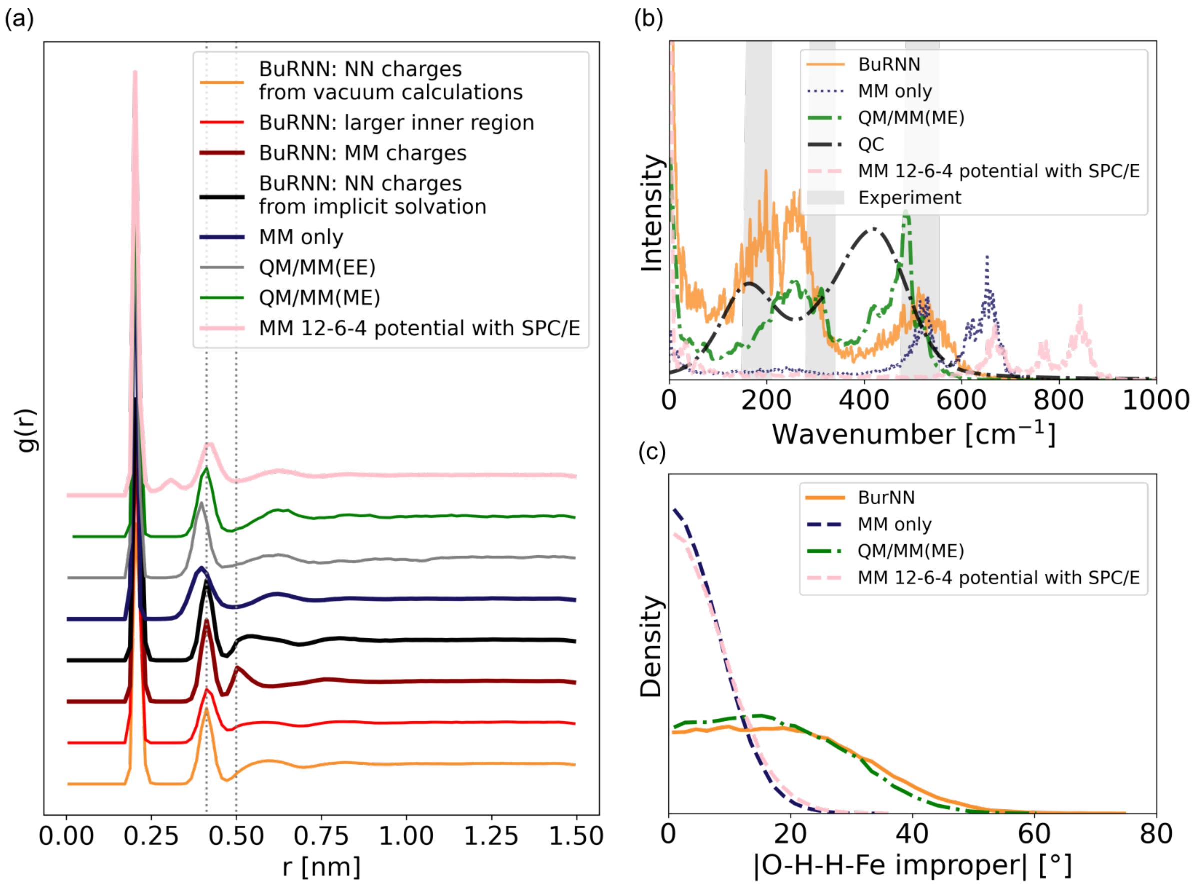

Figure S4: a) Iron-oxygen radial distribution function for different simulations settings. The blue curve shows the radial distribution function for a purely classical simulation (MM only), the orange (red) one for a regular BuRNN simulation (with a larger inner region), in which the partial charges for the inner and buffer region were determined from the QM calculations in vacuum. The black and dark red curves show the radial distribution function for a simulation in which partial charges for the inner and buffer region were estimated from QM calculations in an implicit solvent and assigned according to the classical force field, respectively. Grey and green lines show QM/MM simulations with density functional theory, once using an electrostatic embedding (EE) and once an mechanical embedding (EE), respectively. The pink line shows the radial distribution function obtained with MM 12-6-4 potential with SPC/E. Dotted lines indicated the second BuRNN peak and the cutoff used for the buffer region. Dashed lines indicate the second BuRNN peak and the cutoff used to define the buffer region b) Comparison of power spectra. QC refers to a quantum chemistry calculation of $Fe(H_2O)_{21}$ with the reference method used to generate the training set. training set. Only modes related mostly to the Fe-O bond lengths are shown. The bands were created with a half-peak width of 100 cm^{-1} and for broadening a 1:1 mixture of Gaussian and Lorentzian functions were used. c) Probability distribution of the O-H-H-Fe improper dihedral angle obtained from BuRNN, MM only, QM/MM with EE and the MM 12-6-4 potential with SPC/E simulations.

3) The application presented here is a very special case in which only one atom is included in the inner region. This obviously simplifies the problem by reducing (i) the number of features needed for learning and (ii) the variance of the target quantities. I wonder whether this approach is really scalable to e.g. a solute molecule in water, or any coordination compound with different ligands.

We thank the reviewer for the points raised which are indeed relevant to discuss in the manuscript. We agree with the reviewer that one could assume that a single atom in the inner region reduces the problem considerably and thus also the number of features needed for learning or the variance of the target quantities. However, this is not the case as the model is trained on the coordinates of the inner + buffer region, which consists of about 60 – 70 atoms. The model thus learns the whole system and not just a single atom.

To further address this concern, we have created an additional data set, in which the inner region is represented by the central iron and the first coordinating shell. The buffer region then comprises the second solvation shell. In total, we generated about 6000 data points and used about 5000 for training of new NN models.

The errors are, again, astonishingly small, and comparable to the models trained on the previous data with the smaller inner region. The errors for the models trained on the smaller inner region on approximately the same amount of data is 1.97 kJ/mol (4.73 kJ/mol) MAE (RMSE) for energies and 12.1 kJ/mol/nm (42.3 kJ/mol/nm) MAE (RMSE) for forces. The models trained on the larger inner region have an MAE (RMSE) for energies of 1.97 kJ/mol (7.9 kJ/mol) and for forces 10.3 kJ/mol/nm (22.9 kJ/mol/nm). This experiment clearly shows that the problem we have chosen, i.e., a single iron atom in the inner region, does not simplify the problem and also larger regions lead to similar results. We further would like to stress (as also pointed out by reviewer 1) that these errors are extremely low, about a factor of 2 smaller than what is considered chemical accuracy (1 kcal/mol) and often the target accuracy in many machine learning studies.

These models were further used to run BuRNN simulations. The radial distribution function is plotted for comparison in Figure S4 (previous Figure S3). As can be seen, results are comparable to those obtained from BuRNN with a smaller inner region.

4) The largest difference between BuRNN and MM is in the power spectrum shown in Figure 3. This is compared to experimental FTIR spectra of FeCl3. However, while the FTIR spectra reveal clearly separated bands, the power spectra show a very noisy band around 200 cm-1. In addition, experimental spectra of ref. 47 also contain the effect of Cl ions in the coordination shell, which changes both the symmetry and the reduced mass of the oscillator. I am not sure that the comparison with experiments is clean enough to be used as a validation of the model.

It is true that the experiment is not performed on a single ion in solution, as this would be impossible. Still, we have taken the data for the most dilute solution, of which the authors of the experimental work suggest that the influence of the Cl ions is negligible as they are expected to be far removed from the Fe^{3+} ions. The power spectra from simulation data typically leads to a broad peak as the simulations are of finite length and the thermal noise is significant. We have added a comparison from the QM/MM data for these spectra (as shown in the figures reproduced above) and also computed the vibrational modes from single QM calculations in the local energy minima (included in figure S4c). We consistently find that frequencies observed from BuRNN, QM/MM and a normal mode analysis of QM calculations agree better with the experimentally determined bands at 180, 310 and 500 cm^{-1} than the classical descriptions.

Other points:

5) If I understood correctly, ESP atomic charges are calculated at the QM level on the inner+buffer region. Since this region is quite large, the ESP charges of atoms far from the surface are probably not well fitted. Have the authors checked if the ESP charges of "internal" waters are similar to those of "surface" waters?

Indeed, the partial charges are derived in an ESP approach and this has the risk of leading to irrelevant charges in the inner core. We have tested this in the current work and monitored the charges on the Fe-ion and on the water molecules. Very consistent distributions are obtained. When plotting the atomic charges as function of the distance from the center, we find that very reasonable charges have been derived (see figure S1, also reproduced below). Outliers in the oxygen charges come from configurations in the training set in which the water molecules are oriented with the hydrogen atoms towards the iron. However, for larger systems or for systems in which the solute is not so heavily charged, this may pose a challenge. We have added a “warning” in this respect to the Supporting Information.

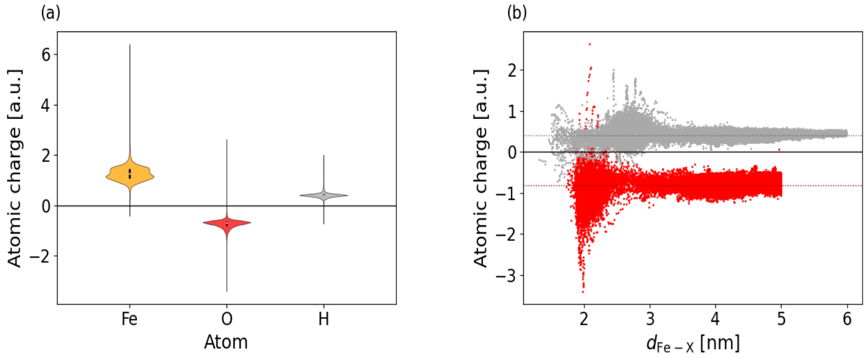

Figure S1: (a) Partial atomic charges of the data set and (b) their dependence on the distance to the Fe atom for oxygen (red) and hydrogen (grey). Dotted lines represent corresponding atomic charges in the SPC water model.

6) In the SI it is stated that the Fe3+ LJ parameters were taken from Zou et al., but in that reference it seems that LJ parameters were derived for Fe in the Heme group. I wonder if this can bias the MM level towards a worse agreement with experiments.

In the context of implementing the 12-6-4 LJ parameters as outlined above, we have double checked the parameters used in the rest of the simulations. To our embarrassment, we discovered that we did not use the parameters as described by Zou et al., but we used the parameters of the IOD set of Li and Merz to begin with. We have clarified this in the SI and thank the reviewer for their comment.

7) Figure S3 compares the RDFs calculated with different definitions for the charges (MM vs NN-implicit vs NN-vacuum). The authors claim that there is an artifact when using MM charges, but it is difficult to notice it from Figure S3, especially since the relevant RDFs are represented with dashed lines.

We have revised the indicated figure and now display the individual RDFs with an offset, which facilitates a comparison. We have furthermore added thin vertical lines to ease the comparison in figure S4a (previous figure S3a). For the indicated simulations, a clear artifact at the distance of 0.5 nm can be observed (see reproduced figure above).

8) The training protocol used here seems to be a very ad-hoc and cumbersome procedure. There is a step in which 21 configurations from a preliminary BurNN MD were extracted and minimized at the QM level, then 4000 configurations are extracted from the minimization trajectories (is this correct?). Then, 4000 (additional?) configurations are obtained by applying SHAKE and removing the water molecules which moved outside the cutoff. Finally, in addition to the adaptive training, ad-hoc configurations with a lower coordination number were generated. I wonder how much this training process is really applicable to more complex systems.

We agree that the generation of the training set is rather cumbersome in the current work. It is the result of a work in progress and not a recommendation on how to generate proper training data in future work. The approach outlined in the supporting information was taken to create ML models that are as accurate as possible such that we can really focus on the new method. We wanted to eliminate any artefacts that could be caused by an ML model not properly trained in certain regions. Once we could be sure that this was not the case, we could focus on the method itself and its validation. If we did this study again, we would not generate the data in the same way as we did, but would generate the data as we described in the main text using adaptive sampling. This is a method that is often used for ML-enhanced MD simulations and was also used by some of us successfully in other studies. We are thus confident that this process will be applicable to “larger systems”. In the main manuscript, we write on page 9:

The training data set is based on QM calculations and can be generated via sampling from MD simulation snapshots of the targeted system and extended using adaptive sampling.

We further emphasize this in the revised manuscript in the SI on page 4:

We emphasize that the procedure outlined above was the result of a continuous process to obtain a sufficiently large training set, such that we could test the BuRNN approach and not the training. For any further applications we are confident that generation of an initial training set that is SHAKEN and filtered up to the buffer region, followed by an adaptive sampling scheme to generate additional conformations as needed will be sufficient.

Additional Questions:

Urgency: Moderate

Significance: Moderate

Novelty: High

Scholarly Presentation: High

Is the paper likely to interest a substantial number of physical chemists, not just specialists working in the authors' area of research?: Yes

We thank the reviewer for judging our work as novel and well-presented and for acknowledging that it will be of interest to a substantial number of physical chemists.

We hope that you will agree with us that we have addressed all the points raised by the reviewer and that you will find our work of interest to the readers of *The Journal of Physical Chemistry Letters.*

We look forward to your response.

Yours sincerely,

Julia Westermayr and Chris Oostenbrink
